# VN Thin Films via MOCVD Using a New Vanadium Precursor: Linking Growth Chemistry to Functional Surface Properties

**DOI:** 10.1002/smtd.202501972

**Published:** 2025-12-18

**Authors:** Jean‐Pierre Glauber, Julian Lorenz, Ji Liu, Marietta Seifert, Volker Hoffmann, Carlos Abad, Detlef Rogalla, Lars Giebeler, Corinna Harms, Michael Wark, Michael Nolan, Anjana Devi

**Affiliations:** ^1^ Institute for Materials Chemistry Leibniz Institute for Solid State and Materials Research Dresden 01069 Dresden Germany; ^2^ Inorganic Materials Chemistry Ruhr University Bochum 44801 Bochum Germany; ^3^ Institute of Engineering Thermodynamics German Aerospace Center (DLR) 26129 Oldenburg Germany; ^4^ Tyndall National Institute University College Cork T12R5CP Cork Ireland; ^5^ Bundesanstalt für Materialforschung und‐prüfung (BAM) 12205 Berlin Germany; ^6^ RUBION Ruhr University Bochum 44801 Bochum Germany; ^7^ Institute of Chemistry Carl von Ossietzky University Oldenburg 26129 Oldenburg Germany; ^8^ Chair of Materials Chemistry Technical University Dresden 01069 Dresden Germany; ^9^ Fraunhofer Institute for Microelectronic Circuits and Systems (IMS) 47057 Duisburg Germany

**Keywords:** catalyst nanoengineering, density functional theory (DFT) simulation, electrochemical nitrogen reduction, metalorganic chemical vapor deposition, precursor chemistries, structure–property correlation, vanadium nitride

## Abstract

Vanadium nitride (VN) is a promising material for many applications, including the electrochemical nitrogen reduction reaction (eNRR). Catalyst nanoengineering enables experimental validation of its predicted eNRR activity, but most VN catalysts are made by using methods that lack precise control. This study introduces a new metalorganic chemical vapor deposition (MOCVD) process for high‐quality, faceted, and crystalline VN thin films. *N*,*N*’‐diisopropylformamidinatovanadate [V(dpfamd)_3_] is identified as a suitable precursor with favorable thermal properties. Using NH_3_ as a co‐reactant yields pure crystalline VN thin films on Si substrates. To investigate structure–property relationships relevant to eNRR, the films are characterized by X‐ray diffraction (XRD), Rutherford backscattering spectrometry combined with nuclear reaction analysis (RBS/NRA), X‐ray photoelectron spectroscopy (XPS), scanning electron microscopy (SEM), and transmission electron microscopy (TEM). Observing NH_3_’s strong influence during growth, first principles density functional theory (DFT) simulations are performed, supporting an energetically favorable decomposition pathway of [V(dpfamd)_3_] to VN with NH_3_ present. Process transfer from Si to conductive Ti substrates, required for in‐depth electrochemical testing, yields VN thin‐film properties similar to those on Si. Preliminary eNRR measurements indicate a potential correlation between faceting and eNRR activity, highlighting the potential of MOCVD‐grown VN thin films for future eNRR applications.

## Graphical abstract

A new vanadium precursor enables metalorganic chemical vapor deposition growth of pure and crystalline VN thin films with tailored orientation. Combined experiments and density functional theory calculations reveal the crucial role of NH_3_ in controlling decomposition pathways and film characteristics. Transferring the process to conductive Ti substrates reveals VN as a promising candidate for nanoengineered electrocatalysts in sustainable nitrogen reduction.

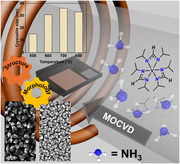

## 1 Introduction

Owing to their distinctive functional properties, transition metal nitrides (TMNs) of Ti, Zr, Hf, V, Nb, Ta, Cr, Mo, and W are appealing for various research fields as they are more thermodynamically stable than their respective parent metals. Their exceptional physicochemical properties include high corrosion resistance,^[^ ^1^ ^]^ hardness, and thermal stability, allowing applications in harsh conditions.^[^ ^2^, ^3^, ^4^ ^]^ Their high electrical conductivity results from a unique electronic structure similar to noble metals.^[^ ^1^, ^5^ ^]^ This makes this material class highly promising for electronic applications.^[^ ^1^ ^]^ In particular, thin films of vanadium nitride (VN) are used as hard coatings,^[^ ^6^, ^7^ ^]^ in (micro‐)supercapacitors,^[^ ^8^, ^9^ ^]^ batteries,^[^ ^10^, ^11^ ^]^ catalysts for biomass valorization,^[^ ^12^ ^]^ absorption materials,^[^ ^13^ ^]^ as well as in electrochemical hydrogen and oxygen evolution reactions (HER, OER).^[^ ^14^ ^]^ Similarly, since HER is a key process for renewable hydrogen production, sustainable pathways for ammonia synthesis are highly desirable to reduce the environmental impact of the Haber–Bosch process, which requires considerable energy input and emits ≈450 million metric tons of CO_2_ annually. Given ammonia's crucial role in fertilizer production for global agriculture, alternative routes, such as electrochemical nitrogen reduction reaction (eNRR), offer promising opportunities to reduce the environmental footprint of ammonia's synthesis.^[^ ^15^, ^16^ ^]^

For this application, TMNs are expected to be particularly suitable, as they are predicted to enable eNRR via the Mars‐van Krevelen (MvK) mechanism, in which nitrogen vacancies serve as active surface sites for N_2_ activation.^[^ ^17^ ^]^ This mechanism may reduce the overall energy barrier for NH_3_ production,^[^ ^18^, ^19^ ^]^ and recently, theoretical research has described VN as both active and selective for the eNRR.^[^ ^20^, ^21^, ^22^ ^]^ Several experimental studies have been conducted to validate the theoretically predicted capabilities.^[^ ^17^, ^23^, ^24^, ^25^ ^]^ Although some of these studies report catalytic activity toward eNRR, others find no ammonia generated from the electrocatalytic process and suggest that structural modifications of the catalyst material are required to unlock the theoretically predicted potential.^[^ ^26^ ^]^

To enhance the activity, efficiency, selectivity, and stability of the catalyst material, various strategies such as heteroatom doping, vacancy engineering, crystal facet (re)arrangement, and heterostructure fabrication can be considered.^[^ ^27^ ^]^ The effect of nanoengineering has been demonstrated by a V_2_O_3_/VN hybrid catalyst, which showed a higher ammonia yield than either the individual oxide or the nitride.^[^ ^28^ ^]^ While theoretical studies suggest that specific facets of VN are advantageous for eNRR characteristics,^[^ ^20^, ^21^ ^]^ a combined theoretical and experimental study indicates that vanadium oxynitrides (VO_x_N_y_) are effective for nitrogen reduction to ammonia.^[^ ^23^ ^]^

Although nanoengineering of the catalyst is a key approach for enabling eNRR on TMNs, the VN catalysts studied so far were either fabricated by nitridation of V_2_O_3_ nanowire arrays,^[^ ^25^ ^]^ VO_2_ nanosheets,^[^ ^24^ ^]^ or via the urea glass method.^[^ ^17^, ^26^ ^]^ While these synthesis approaches can produce the targeted material, they do not provide the nanoengineering capabilities needed to exploit VN in eNRR. Additionally, these materials lack a thin‐film morphology, which could enhance properties such as improved durability, stronger substrate binding for better electrode contact, and enhanced mass transfer.^[^ ^29^ ^]^ Furthermore, employing the catalysts as thin films makes surface‐sensitive structural characterization techniques more effective.

Vapor‐phase deposition methods, such as metal–organic chemical vapor deposition (MOCVD), can produce thin‐film catalysts on a large scale and allow precise tuning of material properties by varying process parameters. Since MOCVD thin film formation relies on the decomposition of the precursor and chemical reactions on the surface, the chemistry of the precursor significantly influences the material's characteristics. Although there are several reports on the CVD of VN, the precursor library is limited mainly to chlorides and amides. Atmospheric‐pressure CVD (APCVD) with VCl_4_ in the presence of NH_3_ yields VN thin films with significant oxide contamination.^[^ ^30^ ^]^

Improved compositional purity was achieved by either changing the co‐reactant to NH(SiMe_3_)_2_^[^ ^31^ ^]^ or by using the vanadium‐amide precursor [V(NMe_2_)_4_] in combination with NH_3_ as the co‐reactant.^[^ ^32^, ^33^ ^]^ Additionally, one carbon‐coordinated precursor, namely [V(C_6_H_6_)_2_], was also reported to form VN at elevated deposition temperatures of 600 °C in the presence of NH_3_, but required a significant excess of the co‐reactant to reduce carbon incorporation into the thin films due to otherwise incomplete precursor decomposition.^[^ ^34^ ^]^

Although volatile alkylamides have been used for MOCVD of VN, their limited thermal stability can be a drawback, as noted in VN growth via atomic layer deposition (ALD).^[^ ^35^, ^36^, ^37^ ^]^ Additionally, the surface reaction pathway of alkylamide‐based precursors is reported to favor the formation of Si–C species, leading to carbon incorporation at the silicon/thin film interface.^[^ ^38^ ^]^ To address these drawbacks, alternative precursor chemistries such as acetylacetonates,^[^ ^39^, ^40^, ^41^ ^]^ alkoxides,^[^ ^42^, ^43^, ^44^, ^45^, ^46^ ^]^ and amidinates^[^ ^47^ ^]^ have been employed.

While the oxygen‐containing precursors are not ideal for the fabrication of VN due to the risk of oxygen incorporation from the ligand,^[^ ^48^ ^]^ the all‐N‐coordinated amidinate [V(dpamd)_3_] offers beneficial properties. Generally, *N*,*N’*‐chelating ligands like amidinates or the related guanidinates and formamidinates offer flexibility for tuning precursor properties, as they can stabilize the metal center through a delocalized π‐electron system while still maintaining sufficient reactivity from their highly reactive M─N bonds. Accordingly, numerous homoleptic and heteroleptic ALD and MOCVD precursors employing these ligand systems have been reported for several transition metals including Ti,^[^ ^49^, ^50^ ^]^ Zr,^[^ ^50^, ^51^ ^]^ Hf,^[^ ^52^, ^53^, ^54^ ^]^ and Mn,^[^ ^55^ ^]^ and were successfully used for the deposition of other TMNs such as NbN,^[^ ^56^ ^]^ ZrN,^[^ ^51^ ^]^ MoN, and WN.^[^ ^57^, ^58^ ^]^

For V, the syntheses of the guanidinate‐ and amidinate‐based precursors have already been reported.^[^ ^59^, ^60^ ^]^ At the same time, the [V(dpamd)_3_] has been used for the ALD growth of vanadium oxides^[^ ^47^ ^]^ and sulfides.^[^ ^60^, ^61^ ^]^ This precursor offers great potential for the fabrication of nitrides as it features a vanadium metal center in the oxidation state +III, and therefore avoids an additional reduction step during the growth of VN.

To the best of our knowledge, there are no reports of this precursor being used for VN thin films. One potential limitation of [V(dpamd)_3_] is the high bubbler temperature of 185–190 °C that is required for sufficient precursor evaporation.^[^ ^60^, ^61^ ^]^ To address this issue, our study attempted to replace the methyl group on the amidinate backbone with a hydrogen atom to produce the formamidinates, as this ligand is known to enhance the volatility of precursors compared to the respective amidinate analogues.^[^ ^62^ ^]^

Herein, we report the synthesis of a new *N*,*N’*‐diisopropylformamidinato complex of vanadium, [V(dpfamd)_3_], validated by ^1^H‐NMR spectroscopy, Fourier transform infrared spectroscopy (FTIR), liquid injection field desorption ionization mass spectrometry (LIFDI‐MS), CHN elemental analysis (EA), and inductively coupled plasma optical emission spectroscopy (ICP‐OES). The thermal properties were investigated by thermogravimetric analysis (TGA) and isothermal thermogravimetric analysis (iso‐TGA). Based on the promising thermal properties of [V(dpfamd)_3_], we utilized this compound for MOCVD of VN thin films on Si and Ti substrates. During process optimization, particular attention was given to the influence of the NH_3_ co‐reactant flow and deposition temperature on thin film properties. Employing a combination of complementary analytical methods, namely X‐ray diffraction (XRD), Rutherford backscattering spectrometry along with nuclear reaction analysis (RBS/NRA), X‐ray photoelectron spectroscopy (XPS), scanning electron microscopy (SEM), high‐resolution transmission electron microscopy (HRTEM), and scanning transmission electron microscopy (STEM), we thoroughly characterized the bulk material and surface properties. This comprehensive analysis facilitated the identification of the essential structural features required for the development of effective eNRR active catalysts. Since the thin film analysis showed a significant influence of the NH_3_ co‐reactant on the grown material, the decomposition of the precursor to VN following a single‐source precursor (SSP) approach was compared with an NH_3_‐assisted pathway using first‐principles density functional theory (DFT) calculations. For the prospective testing of VN as an eNRR catalyst, the process was transferred to conductive Ti substrates, followed by in‐depth characterization of the thin film properties by XRD, glow discharge optical emission spectrometry (GDOES), RBS/NRA, XPS, and HR‐TEM, to ensure comparable film growth as on Si substrates. Preliminary eNRR measurements demonstrate a correlation of VN faceting with ammonium formation. This comprehensive study of VN material synthesis as thin films under moderate preparation conditions, with potential to serve as future eNRR catalysts, is described in this research.

## 2 Results and Discussion

### 2.1 From Precursor Chemistry to VN Thin Films

**Figure 1:**
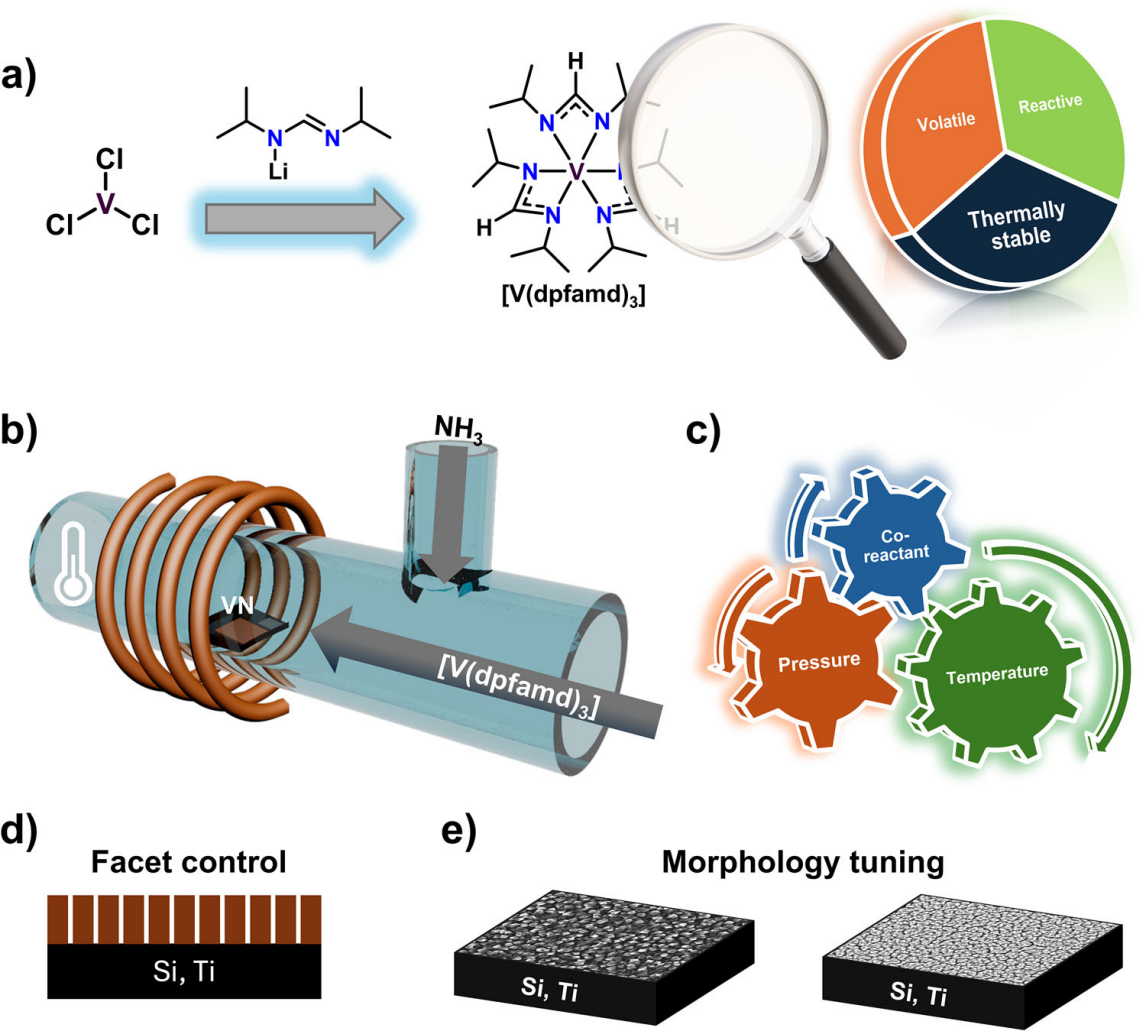
Schematic illustration of the process from precursor design to nanoengineered VN thin films. a) Precursor synthesis and evaluation of key properties. b) Schematic of the MOCVD process, with c) parameter variation enabling d) facet‐controlled growth and e) surface morphology tuning.

Our approach addresses the limited availability of suitable vanadium precursors, which have hindered progress in MOCVD for VN thin films. Specifically, the relationship between precursor chemistry, process parameters, and the structural properties of VN thin films has not been systematically explored, even though such insights are essential for electronic and catalytic applications. Furthermore, despite theoretical predictions that VN is an active catalyst, no experimental studies have investigated faceted VN thin films for eNRR. In this work, the new precursor [V(dpfamd)_3_] was synthesized and evaluated (**Figure** **1**a), then subsequently applied in MOCVD to grow VN thin films on Si and Ti substrates (Figure 1b). By varying deposition parameters (Figure 1c), the crystallographic orientation (Figure 1d) and surface morphology (Figure 1e) were tailored. The resulting structural properties were examined to establish a link with potential catalytic performance. This sequence, from precursor design to nanoengineered thin films, emphasizes the vital role of precursor chemistry in achieving application‐specific material properties.

### 2.2 Precursor Synthesis and Characterization

**Scheme 1:**
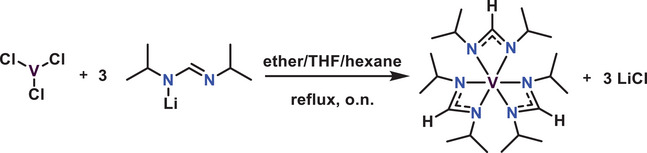
Synthesis pathway for [V(dpfamd)_3_].

**Figure 2:**
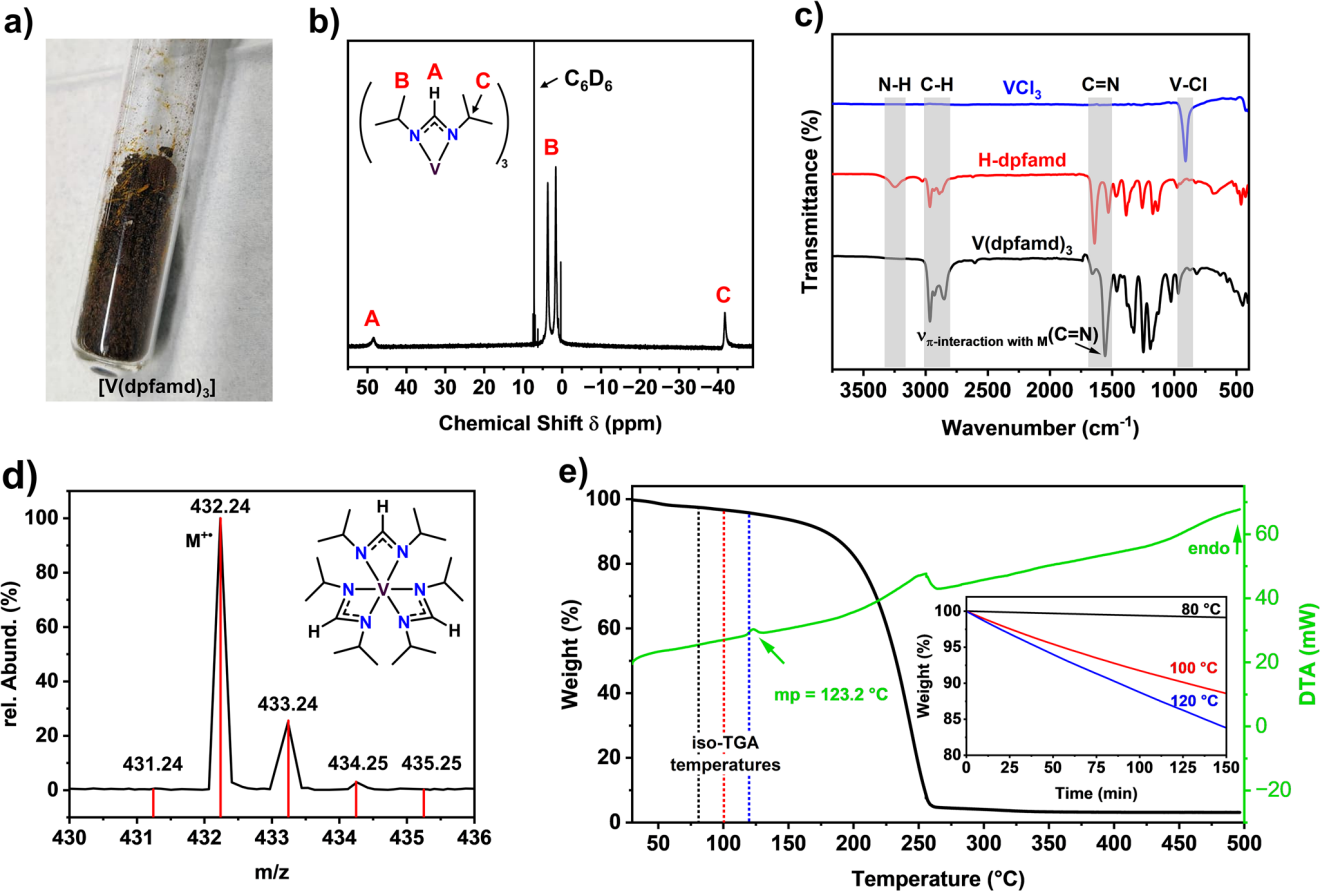
a) Photograph of [V(dpfamd)_3_] after isolation. Precursor characterization: b) Paramagnetic ^1^H‐NMR spectra (C_6_D_6_, 300 MHz) of [V(dpfamd)_3_] with peaks assigned. c) FTIR spectra of [V(dpfamd)_3_] (black), VCl_3_ (blue), and the protonated ligand (red). d) LIFDI‐MS spectra of the molecular ion (M^+•^) peak of [V(dpfamd)_3_] with the calculated isotopic distribution pattern shown in red. e) TGA (black) and DTA (green) of [V(dpfamd)_3_], including iso‐TGA at 80 °C (black), 100 °C (red), and 120 °C (blue), shown in the inset.

Motivated to improve volatility while maintaining the beneficial properties of previously reported [V(dpamd)_3_], including high thermal stability, sufficient reactivity, and a V(III) metal center, we attempted to synthesize the target compound [V(dpfamd)_3_]. This process began by synthesizing the formamidinate (H‐dpfamd) ligand, adopting the previously reported procedure.^[^ ^52^ ^]^ Subsequently, the target complex [V(dpfamd)_3_] was synthesized via a salt metathesis reaction between the in situ prepared lithium formamidinate [Li(dpfamd)] and vanadium trichloride (VCl_3_) in a mixture of ether, tetrahydrofuran (THF), and hexane as depicted in **Scheme** **1**. After stirring at reflux temperature and isolating the crude product, [V(dpfamd)_3_] was obtained as a dark brown solid (**Figure** **2**a). It was purified by sublimation at 100 °C under reduced pressure with a yield of 73%.

To confirm the spectroscopic purity of the synthesized complex, ^1^H‐NMR, FTIR, and LIFDI‐MS analyses were performed, with the results shown in Figure 2 and **Table** **1**, while the elemental composition was confirmed by EA and ICP‐OES (**Table** **2**). Despite the paramagnetic nature of the V(III) complex [V(dpfamd)_3_], the ^1^H‐NMR spectrum in Figure 2b provided an initial indication of the successful synthesis of a spectroscopically pure compound. When expanding the measurement range, three distinct peaks **A**, **B**, and **C** are observed. The proton of the formamidinate backbone **A** appears as a broad peak at a chemical shift of *δ* = 48.45 ppm, while the protons of the *i*Pr groups **B** and **C** appear in a similar range as for the previously reported [V(dpamd)_3_] complex^[^ ^60^ ^]^ at *δ* = 2.61 ppm (br) and *δ* = −41.88 ppm (br), respectively.

**Table 1:** Selected vibrational bands obtained from FTIR measurement from the protonated ligand (H‐dpfamd) and [V(dpfamd)_3_].^[^ ^63^ ^]^

Vibrational bands	H‐dpfamd [cm^−1^]	[V(dpfamd)_3_] [cm^−1^]
*ν*_str_(C─H)	2964–2856	2964–2840
*v*_asym_(*i*Pr)	1458	1452
*v*_sym_(*i*Pr)	1380	1372
*v*_br_(*i*Pr)	1166–1126	1166–1120
*v*_str_(C_R_─N)	1250	1242
*v*_rocking_(CH_3_)	968	962

**Table 2:** Elemental compositions of [V(dpfamd)_3_] from EA (C, H, N) and ICP‐OES (V).

	C [%]	H [%]	N [%]	V [%]
Calc.	58.31	10.49	19.43	11.78
Exp.	57.28	10.49	19.06	11.93

To further validate a successful synthesis, FTIR spectra of the educts VCl_3_ and H‐dpfamd were compared to that of the isolated complex [V(dpfamd)_3_] (Figure 2c). The presence of unreacted ligand or VCl_3_ in [V(dpfamd)_3_] is ruled out because the product spectra neither show the characteristic V─Cl vibration at 902 cm^−1^ nor the broad peak of the N─H vibration at 3244 cm^−1^. For both the ligand and [V(dpfamd)_3_], the characteristic C═N stretching vibrations of a Schiff base (secondary aldimine) appear at *ν *= 1636 cm^−1^ and *ν *= 1656 cm^−1^, respectively. Typically, these bands are accompanied by a second band ≈100 cm^−1^ apart, appearing at *ν *= 1524 cm^−1^ for the ligand and at *ν* = 1550 cm^−1^ for [V(dpfamd)_3_]. The increased transmittance at *ν* = 1550 cm^−1^ in the spectra of [V(dpfamd)_3_] compared to the ligand is explained by an additional and overlapping vibrational band related to π‐interaction with the metal center of C═N. Additionally, characteristic ligand bands are observed in both spectra, further confirming the synthesis of the desired product (Table 1).^[^ ^63^ ^]^

LIFDI‐MS offers significant advantages for analyzing reactive, labile, and sensitive metalorganic precursors compared to commonly employed electron impact mass spectrometry, since it enables ionization under milder conditions.^[^ ^64^ ^]^ The LIDFI‐MS spectra of [V(dpfamd)_3_] show the M^+•^ peak at m/z = 432.24 with an isotopic distribution matching the calculated pattern (Figure 2d), further confirming the isolation of [V(dpfamd)_3_]. Additionally, the purity of the complex was further verified by EA and ICP‐OES measurements, which prove that the elemental compositions match the calculated ones (Table 2).

After confirming the successful synthesis of a spectroscopically pure complex, [V(dpfamd)_3_] was subjected to TGA and iso‐TGA measurements to evaluate its thermal properties for potential use as a precursor in an MOCVD process (Figure 2e). [V(dpfamd)_3_] exhibits favorable evaporation behavior, characterized by a single‐step evaporation with an onset of evaporation at 213 °C following an initial mass loss. The DTA curve displays an endothermic peak at 123.2 °C corresponding to the melting point, and a second peak at 255 °C indicating the completion of evaporation, with a residual mass of 3%, which suggests negligible decomposition. In an MOCVD process, the precursor is vaporized at a constant temperature (in a precursor bubbler) over an extended period. To determine whether [V(dpfamd)_3_] can provide a steady evaporation rate, iso‐TGA was performed at 80, 100, and 120 °C (inset Figure 2d). For all temperatures, [V(dpfamd)_3_] exhibits consistent evaporation behavior over 150 min with evaporation rates of 64.43 µg min^−1^ cm^−2^ (80 °C), 85.21 µg min^−1^ cm^−2^ (100 °C), and 169.29 µg min^−1^ cm^−2^ (120 °C), highlighting it as a promising precursor candidate for chemical vapor phase deposition methods.

### 2.3 MOCVD Process Development and Mechanistic Insights

#### 2.3.1 Growth Characteristics: Arrhenius Plot and Structural Evolution

**Figure 3:**
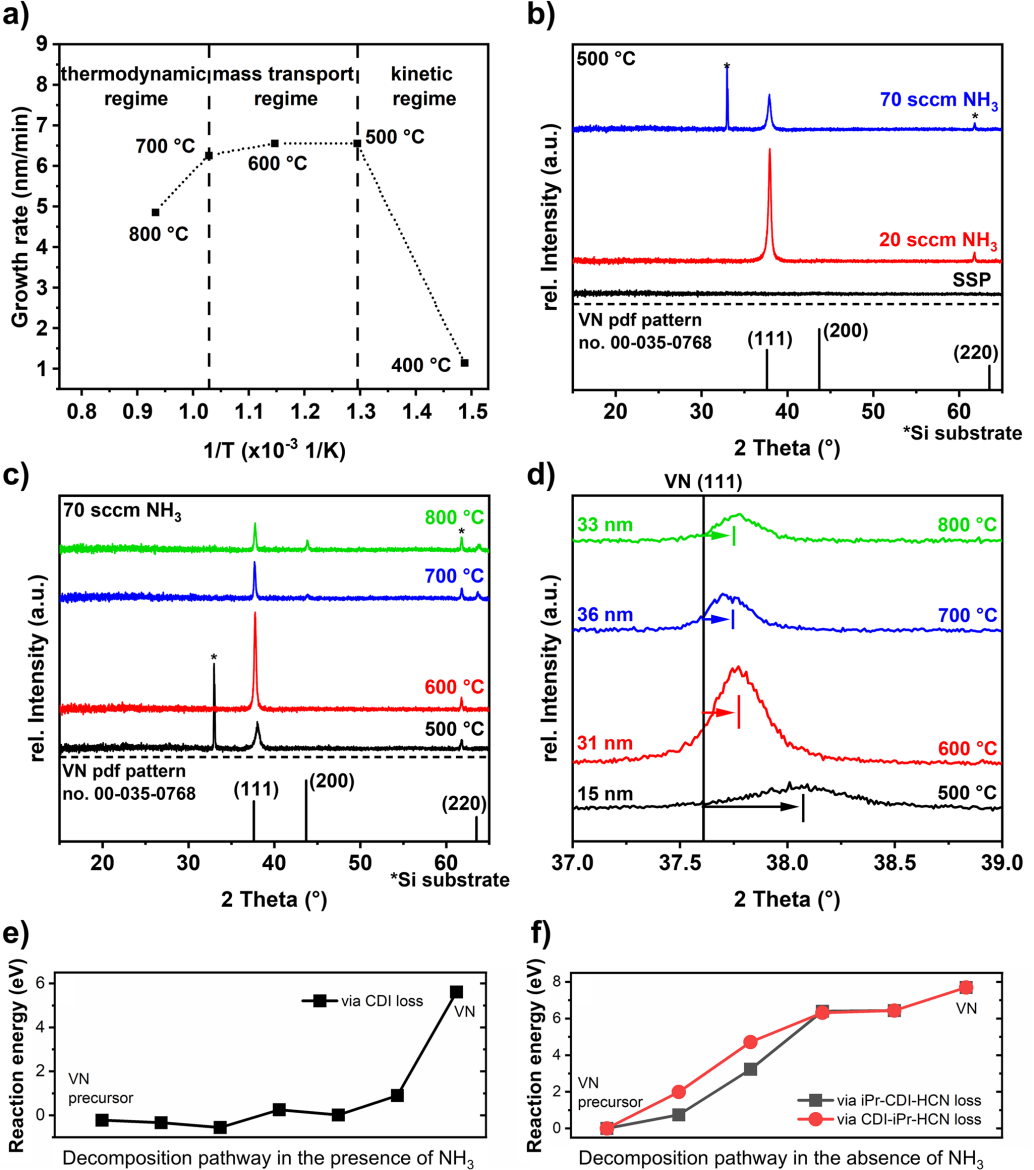
MOCVD growth characteristics: a) Arrhenius plot of the VN film grown on Si substrates using [V(dpfamd)_3_] and 20 sccm NH_3_. b) XRD pattern of VN grown at 500 °C as a function of NH_3_ co‐reactant flow and c) as a function of substrate temperature with a NH_3_ flow of 70 sccm. The XRD reference pattern of VN from the PDF pattern no. 00‐035‐0768^[^ ^67^ ^]^ is shown in black. d) Enlarged view of the 111 reflection, highlighting the shift and grain sizes estimated using the Scherrer equation.^[^ ^66^ ^]^ e) Proposed decomposition pathway of [V(dpfamd)_3_] in the presence of NH_3_ and f) in the absence of NH_3_.

Due to the promising thermal properties of [V(dpfamd)_3_], it was used in MOCVD experiments to grow VN thin films. Before examining the effects of NH_3_ co‐reactant flow and deposition temperature on the material characteristics, N_2_ carrier gas flow, precursor evaporation temperature, and deposition pressure were optimized to 25 sccm, 110 °C, and 10 mbar, respectively (see Experimental Section). Based on the thicknesses estimated from RBS/NRA measurements (**Table** **3**), the typical CVD growth behavior as a function of deposition temperature in the presence of NH_3_ was observed in the Arrhenius plot (**Figure** **3**a). After the kinetic regime, with an increasing growth rate between 400 and 500 °C, a mass transport regime with a steady growth rate was identified between 500 and 700 °C. At higher temperatures above 700 °C, the thermodynamic regime appears characterized by a decreasing growth rate, which could result from either precursor desorption or gas‐phase reactions at elevated temperatures, as is typical of the CVD process.

**Table 3:** Composition derived from RBS/NRA measurements for VN films grown on Si at different NH_3_ co‐reactant flows and substrate temperatures. The samples indicated in bold were further analyzed for their structural properties.

Dep. temp.	V^a)^ [at%]	N^a)^ [at%]	V/N ratio	C^a)^ [at%]	O^a)^ [at%]	d^b)^ [nm]
SSP						
500 °C	26.1	20	1.3	45.4	8.5	–
20 sccm NH_3_						
500°C	41.1	48.8	0.84	8.4	1.7	197
70 sccm NH_3_						
**500 °C**	**41.2**	**48.6**	**0.85**	**6.3**	**3.9**	**100**
600 °C	43.8	51.2	0.86	3.5	1.5	110
**700 °C**	**44.4**	**51.5**	**0.86**	**2.3**	**1.7**	**103**
800 °C	45.4	50.8	0.89	2.2	1.6	81

^a)^ For all concentration values, an error of ±2 at% can be considered;

^b)^ Thicknesses were estimated assuming a VN bulk density of 6.13 g cm^−3^.

To assess the influence of the NH_3_ co‐reactant flow on the composition and the crystallographic structure, thin films grown at 500 °C without NH_3_ flow (SSP conditions), with 20 and 70 sccm NH_3_ were analyzed by RBS/NRA (Table 3) and XRD (Figure 3b). Employing the precursor under SSP conditions, vanadium carbonitride (VC_x_N_y_) is formed with a carbon and nitrogen concentration of 45.4 and 20 at%, respectively. Adding the co‐reactant during growth results in slightly nitrogen‐rich VN thin films with significantly lower carbon content: 8.4 at% (20 sccm NH_3_) and 6.3 at% (70 sccm NH_3_). While the V/N ratio changes only slightly when increasing the deposition temperature from 500 to 800 °C, from 0.85 to 0.89, the carbon content in the thin film decreases from 6.3 to 2.2 at%. Interestingly, the oxygen content in the thin films is below 4 at% across all deposition temperatures, even after handling in ambient conditions. Compared to our previously reported MOCVD growth of ZrN,^[^ ^51^ ^]^ where significant oxygen incorporation from oxidation under ambient conditions was observed, VN is less susceptible to oxidation. This aligns with our recent theoretical study on the surface oxidation of ZrN and VN, which shows a more pronounced oxidation of ZrN.^[^ ^65^ ^]^

Based on XRD data, no reflections were observed for the sample prepared at SSP conditions, indicating the formation of an amorphous thin film. This growth behavior changes in the presence of NH_3_ as the co‐reactant. In the XRD patterns (Figure 3b), the formation of crystalline VN thin films is indicated by the appearance of the 111 reflection of the cubic VN structure at ≈38° 2*θ*, but not the typically expected 200 main reflection. This result supports a preferential growth along the (111) plane. Additionally, increasing the NH_3_ co‐reactant flow from 20 to 70 sccm results in a halved growth rate and a thinner VN‐layer, as indicated by the higher intensity of the substrate reflections. Varying the deposition temperature from 500 to 800 °C at a constant NH_3_ flow rate of 70 sccm causes changes in the VN structure as visualized in the XRD pattern in Figure 3c.

At 500 and 600 °C, only the 111 reflection is observed. Increasing the temperature to 700 °C and above results in the appearance of the 200 and 220 reflections of the cubic VN structure in the space group *Fm*‐3*m*. A shift of the 111 reflections toward lower angles is triggered by the increasing deposition temperature and the different crystallization behavior (Figure 3d). In addition to the enhanced compositional purity of the films obtained at elevated temperatures, these samples also exhibit significantly larger crystallite/domain sizes. Using the Scherrer equation,^[^ ^66^ ^]^ the crystallite or domain sizes of ≈15 nm were roughly estimated at 500 °C, which increased to ≈31 and ≈36 nm at 600 and 700 °C, respectively. At 800 °C, a similar crystallite size of ≈33 nm was obtained, which is consistent with the reduced deposition rate observed at higher substrate temperatures.

#### 2.3.2 Mechanistic Pathways: DFT Simulations of Precursor Decomposition

To understand how NH_3_ affects the MOCVD growth of [V(dpfamd)_3_], gas phase DFT calculations on precursor decomposition were performed with and without NH_3_ as co‐reactant. The computed reaction energies along the decomposition pathway are shown in Figure 3e,f, and the configurations of each elementary step are shown in Figure S1 (Supporting Information). The atomic structures are given in the Supporting Information and a Zenodo repository (https://zenodo.org/records/17495745). These simulations allow the preferred decomposition pathway to be determined, and the energetics of the decomposition chemistry indicate the feasibility of MOCVD at accessible temperatures.

The decomposition of the [V(dpfamd)_3_] precursor without NH_3_ occurs via the loss of two carbodiimide (CDI) ligands (*N*,*N*′‐diisopropylcarbodiimide, C_7_H_14_N_2_), two *i*Pr ligands (C_3_H_8_), and HCN elimination. The overall computed reaction energy for this decomposition process is +7.7 eV. When CDI is released initially, the loss of the first CDI and second CDI ligands has computed energy costs of 1.99 and 2.73 eV, respectively. If *i*Pr is released first, the loss of the first *i*Pr and second *i*Pr ligands has a computed energy cost of 0.74 and 2.49 eV. These considerable positive reaction energies indicate that the decomposition of the [V(dpfamd)_3_] precursor in the absence of NH_3_ is not particularly energetically favored, explaining the observed formation of VC_x_N_y_ under SSP conditions.

In the presence of NH_3_, the computed reaction energy for the first removal of CDI is −0.22 eV, while the energy for *i*Pr removal is 0.64 eV. These energies are considerably lower than those under SSP conditions. Therefore, we propose that the decomposition pathway in the presence of NH_3_ proceeds via loss of CDI ligands, with a slightly negative reaction energy, suggesting continuous introduction of NH_3_ molecules that promote CDI elimination. The computed overall reaction energy is 5.61 eV, which is much less endothermic than the decomposition without NH_3_ but still achievable under MOCVD conditions.

### 2.4 Characterization of VN Thin Films

#### 2.4.1 Surface Composition

**Figure 4:**
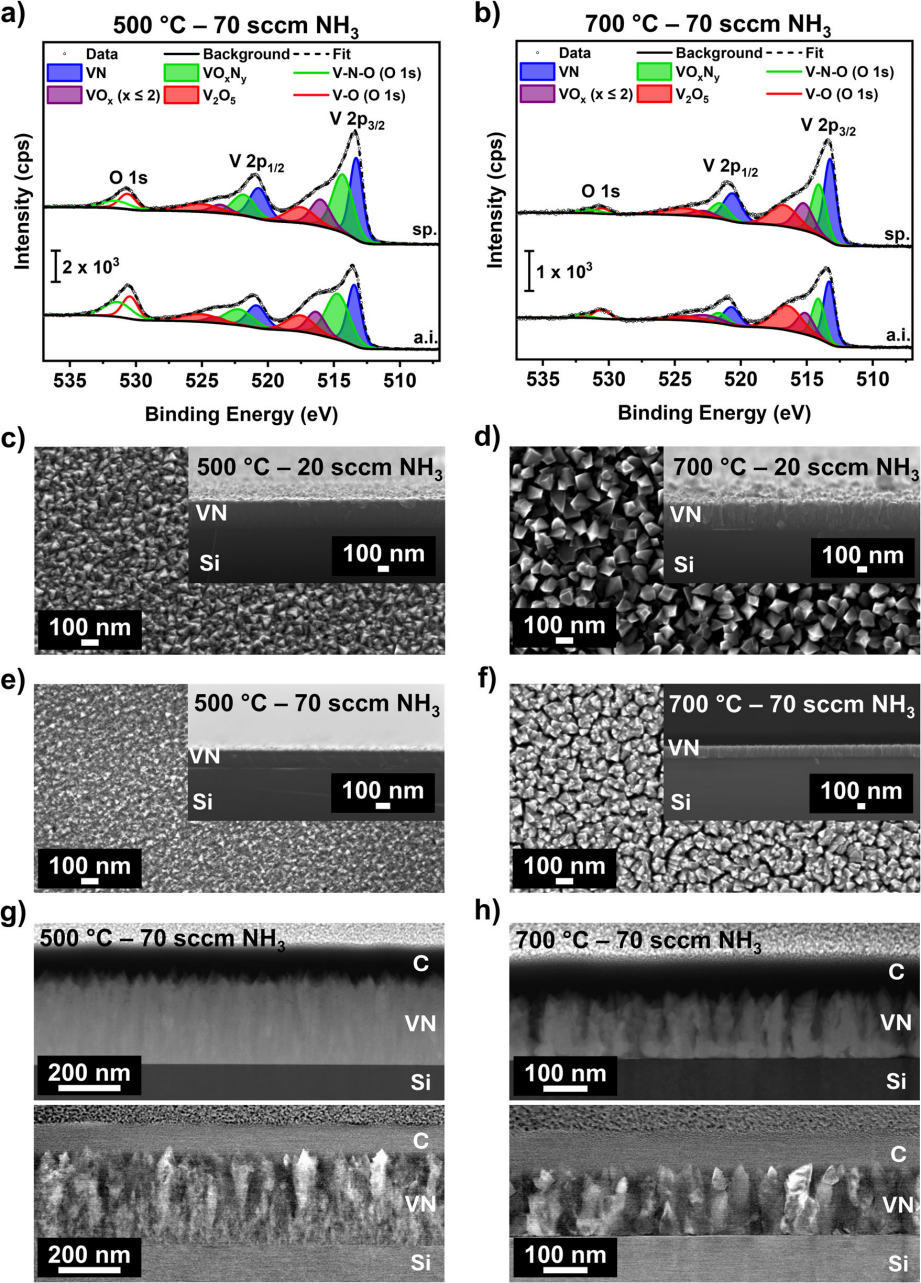
Surface composition analysis: High‐resolution core‐level spectra of V 2p and O 1s of VN grown on Si at a) 500 °C and b) at 700 °C. All measurements were conducted on the as‐introduced (a.i.) surface and after sputtering (sp.). Thin film morphology assessment: c–f) Top view (TV) and cross section (CS) (inset) SEM images (20 kV) of VN as a function of NH_3_ carrier gas flow and deposition temperature: c) 20 sccm NH_3_ at 500 °C, d) 20 sccm at 700 °C, e) 70 sccm at 500 °C, and f) 70 sccm at 700 °C. g,h) STEM images of VN grown on Si with predominant element contrast (top) and predominant orientation contrast (bottom) at g) 500 °C and h) 700 °C.

Compositional analysis of the thin films using RBS/NRA indicates some oxidation due to sample handling in ambient conditions. To examine the nature of surface oxygen species, VN thin films grown at 500 and 700 °C were analyzed by XPS. High‐resolution spectra of the V 2p/O 1s core levels (**Figure** **4**a,b) and the N 1s core levels (Figure S2a,b, Supporting Information) were compared between the as‐introduced samples (a.i.) and those subjected to sputtering (sp.) with Ar^+^ ions.

Before analysis, for all XPS spectra, a charge correction based on the characteristic adventitious carbon (C–C) peak appearing at 284.8 eV^[^ ^68^ ^]^ was conducted (Figure S2c,d, Supporting Information). At both deposition temperatures, the high‐resolution spectra of the V 2p core level feature four different vanadium species that can be assigned to VN, VO_x_N_y_, VO_x_ (*x* ≤ 2), and V_2_O_5_ with clearly separated V 2p_3/2_ and V 2p_1/2_ spin‐orbit components (*Δ* = 7.4 to 7.6 eV). The presence of oxidized species on the surface is often observed for VN and in general, for TMN materials,^[^ ^4^, ^31^, ^69^ ^]^ while structure‐incorporated oxygen like in VO_x_N_y_ has been described as being beneficial for the anticipated use as an eNRR catalyst.^[^ ^23^ ^]^ Additionally, a broadening of the V 2p_1/2_ compared to the V 2p_3/2_ is observed for all features in all spectra due to the Coster–Kronig effect.^[^ ^70^, ^71^, ^72^, ^73^, ^74^ ^]^

Both dominant features are assigned to VN species (513.4 eV), and the peak corresponding to VO_x_N_y_ (514.7 eV) matches literature‐reported values.^[^ ^75^, ^76^ ^]^ Similarly, the obtained binding energies of the vanadium oxide species VO_x_ (*x *≤ 2) and V_2_O_5_ are in accordance with literature‐reported values, with binding energies of 516.3 and 517.5 eV, respectively.^[^ ^17^ ^]^ In proximity to the binding energies of the V 2p core level, the O 1s peak appears, and consists of two species at 531.4 eV and at 530.4 eV that could be assigned to O─V─N^[^ ^76^ ^]^ and O─V^[^ ^77^ ^]^ species, respectively. Upon sputtering, a significant reduction in the intensity of the higher binding energy shoulder in the V 2p spectra and the O 1s peak is observed, indicating the presence of oxidized species primarily at the surface. This observation is in accordance with the findings from bulk compositional analysis via RBS/NRA, revealing a low oxygen content, and with theoretical investigations of the surface oxidation of VN, described as mainly occurring on the surface without oxygen species migrating into the bulk.^[^ ^65^ ^]^

All N 1s core levels depict one main feature at 397.3 eV corresponding to N─V─N species in the VN material,^[^ ^76^, ^78^, ^79^ ^]^ accompanied by two additional features characteristic of air‐exposed nitrides at 396.3 and 398.2 eV that correspond to an oxidized superficial layer (oxynitride) and O─V─N species, respectively.^[^ ^76^, ^80^ ^]^ Since the oxynitride peaks are described as originating mainly from oxidation in air,^[^ ^80^ ^]^ the surface of the sample grown at 500 °C is more oxidized compared to the one grown at 700 °C, well aligning with the results from RBS/NRA. After sputtering, the oxynitride peaks are significantly reduced, supporting the finding that oxidation also occurs mainly on the surface, as reported in the literature.^[^ ^65^ ^]^ At higher binding energies, a broad shoulder at 400 eV appears, which is assigned to C─N species, hinting at an incorporation from precursor decomposition by‐products from the formamidinate backbone. While these C─N species overlap with the C─O species (286 eV) in the C 1s core level spectra (Figure S2c,d, Supporting Information), the detection of V─C species at 282.5 eV further hints at a slightly incomplete precursor decomposition during growth and carbide formation.^[^ ^78^, ^81^, ^82^ ^]^

#### 2.4.2 Thin Film Morphology

After revealing the influence of the deposition temperature and NH_3_ co‐reactant flow on the structure and composition, the effect of these parameters on the thin film morphology was investigated by top view (TV), cross‐section (CS) SEM, and STEM imaging (Figure 4c–h).

Both samples deposited at 500 °C show a growth of triangular crystallites. An increase in the NH_3_ flow from 20 to 70 sccm results in a decrease in crystallite sizes from 20–90 to 10–70 nm. At 700 °C, the NH_3_ carrier gas flow also affects the crystallite shape. It changes from separated triangular and cubic crystals with sizes ranging from 40—140 nm for a 20 sccm NH_3_ flow to agglomerated crystals with diameters ranging from 200 nm up to 620 nm for a 70 sccm NH_3_ flow. The high surface area of the samples grown at 700 °C makes them promising for anticipated use as an eNRR catalyst. Thickness measurements at the CS of the thin films show the same trend as the RBS/NRA estimates. An increasing thickness is found at higher deposition temperatures and at lower NH_3_ flow rates. The measured thicknesses of 275 nm (20 sccm, 500 °C), 300 nm (20 sccm, 700 °C), 130 nm (70 sccm, 500 °C), 170 nm (70 sccm, 700 °C) are larger compared to the values estimated from RBS/NRA which hints to a lower density of the VN thin films compared to the bulk properties. Furthermore, CS SEM images indicate a faceted growth of the VN thin films, which was further confirmed by TEM analysis for the films grown at 500 and 700 °C with an NH_3_ flow of 70 sccm (Figure 4g,h).

For both deposition temperatures, STEM revealed faceted, pillar‐like growth with different crystallite orientations and an even element distribution throughout the thin film, evident from STEM images (Figure 4g,h) with predominant orientation contrast (bottom) and predominant element contrast (top), respectively. Although it is not possible to distinguish V and N atoms in EDX measurements in the low‐energy region at ≈0.5 keV due to overlapping peaks, the even distribution of the other elements throughout the whole film is confirmed by TEM‐EDX measurements at the surface, middle, and substrate interface region (Figure S3, Supporting Information). Additionally, a smooth interface is formed between the native silicon oxide of the Si substrate and the VN thin film, as visible from the HRTEM images in Figure S4 (Supporting Information). Compositional analysis by XPS (Figure 4a,b) revealed the presence of surface oxide species that are reduced upon sputtering, suggesting the formation of a thin surface oxide layer and the absence of oxidation in the bulk. The STEM images of both films confirm this observation: a thick oxide layer would be apparent at different contrast levels, but it is not visible in the images.

### 2.5 VN Thin Film Growth on Titanium Substrates

**Figure 5:**
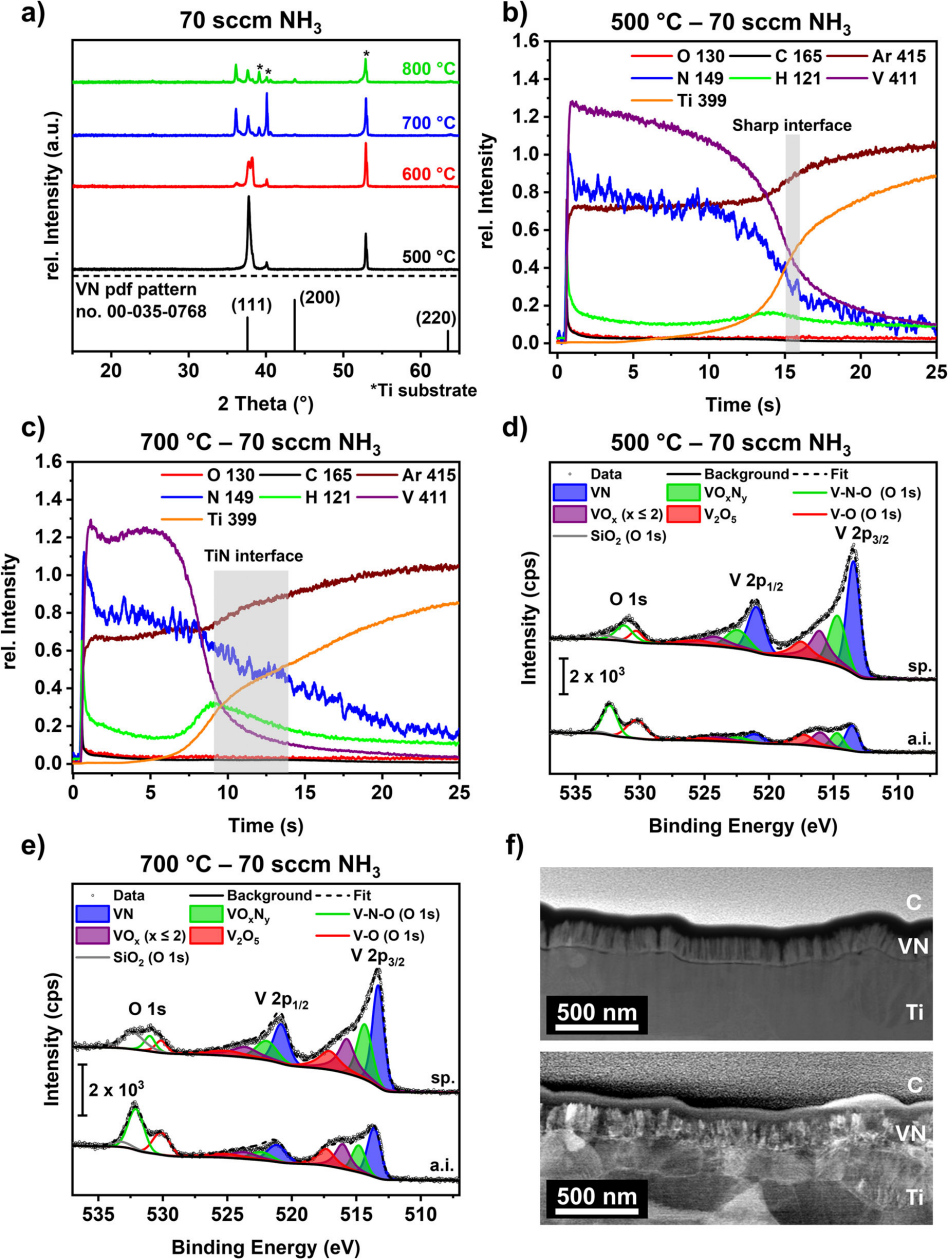
a) XRD patterns of VN deposited on Ti substrate at different deposition temperatures showing the presence of a Ti_x_N_y_‐interlayer as detailed in Figure S5b (Supporting Information). The reference XRD pattern of VN from the PDF pattern no. 00‐035‐0768^[^ ^67^ ^]^ is shown in black. b,c) GDOES measurements of the VN thin film deposited on Ti at b) 500 and c) 700 °C. d,e) High‐resolution core level spectra of V 2p/O 1s of VN grown on Ti at d) 500 °C and e) at 700 °C. All measurements were conducted on the as‐introduced (a.i.) surface and after sputtering (sp.). f) STEM images of VN grown at 700 °C on Ti substrates with predominant element contrast (top) and predominant orientation contrast (bottom).

Since the process optimization and thin film characterization show promising properties for investigating this material as an eNRR catalyst, including crystalline material of high compositional purity with a high surface area and faceted growth, the process was transferred to conductive Ti substrates, which are necessary for electrochemical analysis. Structural analysis using XRD (**Figure** **5**a) shows a trend similar to that on Si substrates, with a more complex pattern due to reflections from the underlying Ti substrate (Figure S5a, Supporting Information). While at 500 °C, only the VN 111 reflection appears near the 002 reflection of the Ti substrate, at higher deposition temperatures, reflections from the (200) and (220) planes are observed. Furthermore, at 700 and 800 °C, additional reflections corresponding to the nitridation of the Ti substrate to both TiN and Ti_2_N (Figure S5b, Supporting Information) are observed, which suggests the formation of an interface between the substrate and the thin film. To verify this Ti_x_N_y_‐based layer, GDOES measurements (Figure 5b,c) of the films grown at 500 and 700 °C were conducted. For the sample grown at 500 °C, a sharp decrease in both V and N signals is observed just before the emergence of Ti substrate species. Conversely, the sample grown at 700 °C displays a sharp reduction in the V signal, while the N signal decreases more gradually as the Ti signal begins to increase. This profile suggests a less‐defined interface between the VN film and Ti substrate, pointing to the formation of a Ti_x_N_y_ interface. RBS/NRA analysis of the films grown on Ti at 500 and 700 °C quantifies a similar composition as the films grown on Si substrates, with V 41.1 at%, N 48.7 at%, C 6.4 at%, O 3.9 at%, and V 44.4 at%, N 51.6 at%, C 2.3 at%, O 1.7 at%, respectively. Furthermore, no Ti was found in the thin film by RBS/NRA measurements. This result indicates that the Ti_x_N_y_ species, which were visible in the XRD and GDOES measurements, were formed directly at the interface between the substrate and the film and that there was no migration of Ti atoms from the substrate into the VN thin film.

This assumption was further supported by XPS analysis of the samples grown on Ti substrates, as no Ti was detected for the pristine samples and the same samples after sputtering for both deposition temperatures. The survey spectra (Figure S6a,b, Supporting Information) reveal the presence of Si impurities, possibly associated to SiO_2_ particles from the reactor's (brittle) heating mantle. As the respective peak diminishes after sputtering and no Si was detected in RBS/NRA, the incorporation of Si into the thin film is excluded. Assignment of respective features of the high‐resolution XPS spectra (V 2p/O 1s (Figure 5d,e), N 1s (Figure S6c,d, Supporting Information) and C 1s (Figure S6e,f, Supporting Information) was identical to the analysis of the VN films grown on Si substrate (see above) except that the O 1s spectra required a fit with a third component at higher binding energies of ≈533.2 eV, which is often related to adsorbed water or organic oxygen.^[^ ^83^ ^]^ Although other characterization techniques showed comparable compositions and structures for VN films grown on either Si or Ti substrates, XPS revealed distinct differences in surface composition.

First, the C 1s spectra (Figure S6e,f, Supporting Information) of pristine samples showed no indication of carbide formation at either temperature. After sputtering carbide features at a binding energy of ≈282.5 eV were observed in both films, with the effect more pronounced in the 500 °C sample.

Second, surface oxidation is more pronounced for VN on Ti than for Si analogues, although RBS/NRA showed similar compositions. This result is related to a higher content of VO_x_ and components compared to VN and VO_x_N_y_, as well as a greater oxygen content in the O 1s spectra (Figure 5d,e). This is possibly influenced by the increased surface area and, consequently, facilitated ambient surface oxidation of rougher Ti substrates compared to smooth Si surfaces, as evident in TEM in Figure 5f. Also, the intensities of the observed oxygen components are reversed. While for VN films on Si the oxide component (O─V) at ≈530.4 eV, related to V oxide species, is more intense, the second component, associated with V─N─O or, more generally, non‐stoichiometric oxygen (e.g., hydroxides), is more pronounced in the case of VN on Ti. As the N 1s spectra of both variants show comparable nitride and oxynitride composition (Figures S2a,b,S6c,d, Supporting Information), the different intensities seem not to be related to a pronounced oxynitride formation. Instead, a more defect‐rich oxygen‐containing phase might account for the intense component at 532.3 eV. Note that SiO_2_ impurities as well as organic O (although no equivalent feature occurred in the C 1s spectrum) can contribute to this signal but both do not explain it solely. Moreover, Ti will form a passivating TiO_2_ layer on the surface. This oxygen can diffuse during the MOCVD process in response to the energy incident on the surface and may result in enhanced surface oxidation of the VN films, particularly on Ti substrates.

Third, the C─N component at ≈400.2 eV in the N 1s spectra is more pronounced in the case of VN samples on Ti, especially for films deposited at 500 °C. This component can be related to fragments from the precursor decomposition (Figure S1, Supporting Information) that are incorporated into thin films, and thus hints at slightly different MOCVD growth characteristics on Ti compared to Si.

On the other hand, the effect of Ar^+^ ion sputtering on the Ti samples is comparable to that on the Si analogues, with oxide components being drastically reduced, demonstrating the impact of ambient surface oxidation.

Similar to VN on Si, the film grown on Ti consists of facets with different crystal orientations (Figure 5f, bottom), and an even element distribution through the whole film was obtained by applying predominant element contrast during the STEM measurement (Figure 5f, top) as well as demonstrated by EDX measurements (Figure S7a, Supporting Information). Additionally, EDX confirmed that XPS findings showed no migration of Ti species from the substrate into the surface (Figure S7b, Supporting Information). The absence of a significant oxide layer on the surface, as hinted by XPS analysis, is confirmed by the absence of any surface layer with a different contrast in the STEM images. Furthermore, the high surface roughness of the Ti substrate is apparent, highlighting one strength of the employed MOCVD technique: its ability to coat such complex structures evenly. Between the VN thin film and the Ti substrate, an interface layer is formed according to a nitridation of the Ti surface at elevated temperatures as shown by XRD and GDOES.

Proof‐of‐principle preliminary eNRR measurements were performed in 0.2 m H_2_SO_4_ electrolyte on two VN thin film samples deposited at either 500 °C (VN‐500) or 700 °C (VN‐700) on Ti substrates to demonstrate the capability of the developed thin films for eNRR application; results are shown in Figure S8 (Supporting Information). The samples were selected because they differ in crystallinity and oxygen content (cf. Figure 5), where both parameters have been previously shown to have an effect on the eNRR activity.^[^ ^17^, ^21^ ^]^ Our preliminary eNRR data showed no activity in the case of the VN‐500 sample possessing a dominant (111) faceting in agreement with theoretical predictions, where this (111) facet has been shown to decompose at NRR‐relevant potentials.^[^ ^21^ ^]^ The VN‐700 sample containing the theoretically active (200) facet, among others, showed instead greater ammonium formation in N_2_‐experiments compared to background Ar‐experiments with production rates of 20–30 pmol s^−1^ cm^−2^ at three selected potentials of −0.2, −0.4, and −0.6 V. Thus, our preliminary eNRR work is in alignment with theoretical predictions. It demonstrates that our MOCVD synthesis of VN can be used to control VN material properties relevant to eNRR. However, we note these preliminary measurements do not prove genuine eNRR activity, as isotope‐labelled ^15^N_2_ turnover experiments are necessary. In addition, stability investigations are required to exclude non‐catalytic decomposition as the source of ammonium formation.^[^ ^26^ ^]^ However, both are beyond the scope of this work, which focuses on the functional surface properties of the VN thin films.

## 3 Conclusion

A new precursor, [V(dpfamd)_3_], demonstrates high suitability for MOCVD of vanadium‐containing thin films. Complementary analytical methods confirm its successful synthesis and spectroscopic purity, while TGA and iso‐TGA reveal desirable thermal behavior, with single‐step, uniform evaporation. Using this precursor in the MOCVD process yields high‐quality, crystalline VN thin films with a preferred (111) orientation, as verified by RBS/NRA, XPS, and XRD.

The presence of NH_3_ as a co‐reactant decreases carbon incorporation to 2.2 at%, and DFT simulations confirm an energetically favored NH_3_‐assisted decomposition pathway. RBS/NRA shows low bulk oxygen levels (<5 at%), whereas XPS indicates surface oxides confined to the topmost layers. The NH_3_ flow significantly influences surface morphology at elevated temperatures, ranging from isolated crystallites (20 sccm) to larger agglomerates (70 sccm). On Ti substrates, VN films exhibit similar properties but form Ti_x_N_y_ interfacial layers, and show increased surface oxidation likely due to higher surface roughness. Overall, this MOCVD process enables precise control over the VN thin‐film properties, providing a robust platform for experimentally validating theoretical predictions of eNRR catalysis. Preliminary eNRR measurements indicate a potential correlation between faceting, controlled by MOCVD deposition parameters, and eNRR activity, which requires further experimentation and will be the subject of follow‐up studies.

## 4 Experimental Section

### General Working Procedure

Reactions involving moisture and air‐sensitive compounds were either conducted using Schlenk techniques with either a conventional vacuum/argon line or inside an Ar‐filled (Air Liquide, 99.998% purity) MBraun Labmaster 120 glovebox. Before using the solvents for the synthesis of the vanadium complex, high‐performance liquid chromatography grade solvents were dried and purified in an MBraun solvent purification system and stored over molecular sieves (4 Å). For the ligand synthesis of H‐dpfamd, the procedure reported elsewhere was used.^[^ ^52^ ^]^ VCl_3_ (97%) and n‐BuLi (1.6 m in hexane) were purchased from Merck Chemicals.

### Synthesis of [V(dpfamd)3]

H‐dpfamd ((2.5 g; 19.50 mmol)) was dissolved in 45 mL of dry ether, and 12.19 mL of n‐BuLi (1.6 m in hexane) was added dropwise at 0 °C. After stirring at room temperature, a suspension of VCl_3_ in 45 mL dry THF was added while maintaining the temperature at 0 °C. The resulting dark violet suspension was stirred at reflux temperature, and subsequently, the solution was separated from the precipitated LiCl via filtration using a Whatman filter. Removal of all volatiles under vacuum followed by sublimation at reduced pressure (10^−3^ mbar) at 100 °C, yielded 2.06 g of a dark brown solid (yield: 73%). To ensure consistency in scalability for [V(dpfamd)_3_] to be used in MOCVD experiments, the synthesis was repeated three times with comparable yields (65%–73%). ^1^H NMR (300 MHz, [D_6_]benzene, 25 °C, TMS): *δ* = 48.45 ppm (br, (*i*PrN)_2_C─**H**), 2.61 ppm (br, N─CH─(C**H**_3_)_2_), −41.88 ppm (br, N─C**H**─(CH_3_)_2_); LIFDI calculated m/z (%) of M^+•^: 431.2 (0.3), 432.2 (100), 433.2 (25.6), 434.3 (3.1), 435.3 (0.2); found m/z (%) of M^+•^: 431.24 (0.5), 432.24 (100), 433.24 (24.9), 434.25 (2.8), 435.25 (0.2); FTIR (ATR, solid), *ν* (cm^−1^) (vibration): 2964–2840 (*ν*_aliphatic str._(C─H)),1656 (*ν*_str._(C═N)), 1550 (*ν*_str._(C═N), *ν*_π‐interaction with M_(C═N)), 1452 (*ν*_asym._(*i*Pr)), 1372 (*ν*_sym._(*i*Pr)), 1242 (*ν*_str._(C_R_─N)), 1166–1120 (*ν*_br._(*i*Pr)), 962 (*ν*_rocking._(CH_3_)); EA calculated for C_21_H_45_N_6_V (%): C 58.31, H 10.49, N 19.43; found (%): C 57.28, H 10.49, N 19.06; ICP‐OES calculated for C_21_H_45_N_6_V (%): V 11.78; found (%): 11.93.

### Precursor Characterization

To ensure air and moisture‐free conditions, the samples for analysis (e.g., NMR, FTIR, EA, and TGA) were prepared inside an argon‐filled MBraun 100 glovebox. For NMR spectroscopy, the NMR solvents from Millipore were stored over activated molecular sieves (4 Å) after degassing and drying. NMR spectra were recorded on a Bruker Avance DPX 300 spectrometer with an extended measurement range to 202.33 ppm and 32 scans. Before analyzing the spectra with MestReNova v10.0.2‐15465 software from Mestrelab Research S.L., the solvent signal (C_6_D_6_) at 7.160 ppm was used as a reference. FTIR measurements were performed using a Perkin Elmer Spectrum Two with a UATR two diamond crystal unit inside an argon‐filled glovebox (MBraun 100). TOF‐MS LIFDI+ measurements were done on a Q‐TOF Premier Micromass mass spectrometer from Waters, Manchester, United Kingdom, where the standard ESI source was replaced with a LIFDI source from Linden CMS, Weyhe, Germany. Approximately 40 nL of a filtered solution of [V(dpfamd)_3_] in toluene was injected into the instrument without breaking the inert conditions by using a fused silica capillary. The spectra of [V(dpfamd)_3_] were referenced to the toluene solvent peak at m/z = 92.05. For EA measurements, the UNICUBE (Elementar) in the CHN analysis mode was used after calibration with a certified acetanilide (Säntis Analytical AG). Vanadium analysis was performed using an ICP‐OES instrument, the iCAP6500 Duo View, from Thermo Fisher Scientific. Before analysis, four parallel weights of 40 mg were dissolved in a mixture of 0.5 mL deionized water and 2 mL nitric acid (65% p.a., Merck). These digestion solutions were weighed to a total mass of 400 g and diluted with deionized water. Every solution was measured four times in bracketing mode. The method was calibrated using a commercial single‐element standard solution (1 g L^−1^ vanadium, Thermo Fisher Scientific). Assessment of thermal properties was conducted at atmospheric pressure in a 90 sccm N_2_ (Air Liquide, 99.998%) carrier gas flow using a PerkinElmer STA 6000 for TGA and a Netzsch STA 409 PC LUXX for iso‐TGA inside an Ar‐filled Plexiglas drybox. For TGA measurements, 16.2 mg of [V(dpfamd)_3_] was heated at a ramp rate of 5 K min^−1^ from 30 to 500 °C, and for each isothermal TGA measurement at 80, 100, and 120 °C, ≈30 mg of [V(dpfamd)_3_] was used.

### MOCVD Process Development

MOCVD of VN using [V(dpfamd)_3_] was conducted in a custom‐built horizontal cold‐wall reactor. The co‐reactant NH_3_ (99.999%) was further purified with a gas purifier tube from Entegris (ENTEGPUS35FYX04R00) before being introduced into the reactor. Both the 1 × 1 cm Si(100) and 1.5 × 1.5 cm Ti substrates were rinsed and cleaned in an ultrasonic bath by HPLC‐grade acetone, isopropanol, and water, dried with Ar (Air Liquide, 99.999%). To optimize the process, various precursor evaporation temperatures between 60 and 110 °C were initially tested, and the N_2_ (Air Liquide, 99.999%) carrier gas flow was varied between 15 and 25 sccm. Subsequently, the deposition pressure was varied between 1 and 10 mbar, resulting in optimized conditions: a precursor evaporation temperature of 110 °C and a N_2_ carrier gas flow of 25 sccm at a deposition pressure of 10 mbar. For assessing the influence of deposition temperature and the NH_3_ co‐reactant gas flow on VN thin film properties, these deposition parameters were varied between 400 to 800 °C and 0 to 70 sccm, respectively, while maintaining a constant deposition time of 30 min. Two hundred milligrams of the precursor was placed into the bubbler that enabled reproducible MOCVD growth of VN five times.

### Thin Film Characterization

XRD was measured using a Bruker D8 Advance diffractometer employing Cu‐K_α_ radiation (*λ* = 1.5418 nm) in Bragg–Brentano (*θ*–2*θ*) geometry over a range of 15° to 65° with an acceleration voltage of 60 kV and a heating current of 30 mA. By employing the position and FWHM of the 111 reflection from the measured VN diffraction pattern in the Scherrer equation,^[^ ^66^ ^]^ crystallite sizes were estimated using the Scherrer constant for cubic (111) reflections (*K* = 0.8551).^[^ ^84^ ^]^ For RBS/NRA, the samples were measured at the RUBION facility (Ruhr University Bochum, Germany) using a 4 MV tandem accelerator; for RBS, a 2 MeV ^4^He^+^ beam with an intensity of 40–50 nA; and for NRA, a 1 MeV deuteron beam. For both measurements, an incident beam at a tilt angle of 7° was applied, and a silicon detector was used to detect the emitted protons; for the detection of backscattered particles, a silicon detector with a resolution of 16 keV at an angle of 160° was employed. Data analysis, including stoichiometry calculations from the RBS and NRA data, was done using the SIMNRA program.^[^ ^85^ ^]^ VN grown on Si was subjected to XPS measurements on a PHI 5000 VersaProbe II instrument with Al K_α_ photon radiation of 1486.6 eV. For the overview survey scans, a step width of 0.5 eV and a pass energy of 187.5 eV were employed, while for the core level scans of the V 2p/O 1s, N 1s, and C 1s, a reduced step width and pass energy of 0.05 and 23.5 eV were used, respectively. The measurements were performed under vacuum (3 × 10^−6^ mbar) with an XPS beam (200 µm diameter) at a tilt angle of 45°. First, the survey spectra of the as‐introduced samples were recorded, and based on the peak intensities of interest, the number of scans was adjusted for the respective core‐level spectra. Subsequently, the exact measurements were conducted after sputtering the surface (2 keV, 2 × 2 mm^2^, 60 s). Analysis of the peaks was done with Casa XPS^[^ ^86^ ^]^ (v2.3.26PR1.0), after introducing a Shirley background and referencing all spectra to the adventitious carbon (C–C) peak at 284.8 eV.^[^ ^68^ ^]^ XPS of VN samples on Ti substrates was performed on an ESCALAB 250 Xi instrument (Thermo Fisher) utilizing a monochromatized Al K_α_ photon radiation (1486.6 eV). Survey spectra were measured with step widths of 1 eV at a pass energy of 100 eV and a dwell time of 10 ms where three scans were conducted. High‐resolution core level spectra of V 2p/O 1s, N 1s, and C 1s (10 scans for V 2p/O 1s as well as N 1s and 5 scans for O 1s spectra) were measured with step widths of 0.02 eV at a pass energy of 10 eV and dwell time of 50 ms. A flood gun was used to compensate for the charging effects of potentially oxidized surfaces. Sputtering with Ar^+^‐ions (2 keV, 3 × 3 mm^2^) was performed for 60 s. Peak deconvolution analysis was performed after referencing all spectra to the adventitious carbon (C–C) peak at 284.8 eV using a product of Gaussian and Lorentzian peak shapes and with a smart background in the Avantage software (version 5.9925). Fitting procedures for VN‐Si and VN‐Ti samples were aligned by adjustment of constraints. The morphology of VN was investigated by imaging the surface (TV) and the CS with a JEOL JSM‐7200F scanning electron microscope coupled with an SE, BSE, in‐lens SE, and EDX detector. For all images, an acceleration voltage of 20 kV and a working distance of 10 mm were used. Crystallite sizes and thicknesses from the SEM images were measured using Fiji Image J,^[^ ^87^ ^]^ and the contrast of all SEM images was enhanced by employing the enhance local contrast function of this program. A detailed analysis of the sample morphology was performed by transmission electron microscopy (TEM). A Tecnai F30 (FEI Company, Hillsboro, OR, USA) was used to image cross‐sections of the thin films by STEM in different contrast modes. In addition, HRTEM images were recorded. Energy‐dispersive X‐ray analysis (EDX, Octane T Optima, EDAX Company, Mahwah, NJ, USA) was performed in the TEM for compositional analysis. The TEM lamellas were prepared in a focused ion beam device (Helios 5 CX, Thermo Fisher Scientific, Waltham, MA, USA), after sputtering a thin carbon layer onto the VN thin film to enhance surface conductivity, protect the surface from ion‐beam damage, and improve imaging quality. This measure was only done for the respective TEM samples. GD‐OES depth profiles were measured in the joint GDOES lab of BAM Berlin and IFW Dresden, using a GDA750 HR (Spectruma Analytik GmbH, Hof, Germany) spectrometer. The samples were sputtered in radio‐frequency mode at 6.78 MHz, 500 V anode voltage, and 2.7 hPa Ar pressure. A modified universal sample unit of Spectruma was used, in which the samples do not act as seals to the atmosphere. This sputtering source was of the Grimm type with a 2.5 mm diameter anode and used water cooling from the backside. All measurements were performed at room temperature (RT).

### Density Functional Theory (DFT) Studies

All the calculations were performed on the basis of periodic spin‐polarized DFT within a plane wave basis set and projector augmented wave formalism,^[^ ^88^ ^]^ as implemented in the Vienna ab‐initio simulation package (VASP 5.4) code.^[^ ^89^ ^]^ The generalized gradient approximation with the Perdew–Burke–Ernzerhof (PBE) parameterization was used for the exchange‐correlation functional.^[^ ^90^, ^91^ ^]^ Five valence electrons were used for V, 5 for N, 4 for C, and 1 for H. The plane wave energy cutoff was set to be 400 eV. The convergence of energy and forces was set to be 1 × 10^−4^ and 1 × 10^−3^ eV Å^−1^, respectively. The precursors and decomposition products were simulated within a 30 Å × 30 Å × 30 Å box, and the Gamma point sampling was employed during the calculations. Gaussian smearing with a width of 0.2 eV is applied. The gas‐phase reactions of the proposed precursor [V(dpfamd)_3_] decomposition pathway, both with and without NH_3_ were modeled. The gas‐phase reaction effectively and accurately represented the MOCVD process.

### Electrochemical Experiments

eNRR measurements were performed in a commercial half‐cell setup (FlexCell, Gaskatel) in freshly prepared 0.2 m H_2_SO_4_ electrolyte equipped with a commercial gas purifier (Agilent OT3‐4) and mass flow controller (SFC5500, Sensirion). A reversible hydrogen electrode (mini‐RHE, Gaskatel) and a coiled PtIr‐wire were used as reference and counter electrode, respectively. The cell and all components were thoroughly cleaned with water and dried in an oven. The measuring protocol (Figure S8a, Supporting Information) included initial cyclic voltammetry (CV) measurements and an activation routine by CV to remove the surface oxide layer on the nitride material.^[^ ^92^ ^]^ Dynamic eNRR (d‐NRR) conditions were applied, where the potential was stepped from +0.6 V (10 s) to −0.6 V (10 s) for 2 h (360 repetitions of both steps). Several samples for quantitative analysis by ion chromatography (IC) were taken after different steps in the protocol (indicated by a red arrow) to exclude possible contaminants on the sample's surface from the determined production rates after turnover measurements. All measurements were performed either in Ar‐saturated (background) or N_2_‐saturated (eNRR) electrolyte with a new sample and a gas flow of 10 mL min^−1^. IC was applied for quantification of formed ammonium (NH_4_^+^) in the 0.2 m H_2_SO_4_ electrolyte (Figure S8b, Supporting Information) with a limit of quantification of 2 µg L^−1^ (see the method development in Bragulla et al.,^[^ ^93^ ^]^ which was further improved in follow‐up work).

## Conflict of Interest

There are no conflicts to declare.

## Supporting information



Supporting Information

## Data Availability

The data that support the findings of this study are available from the corresponding author upon reasonable request.
