# Supplementary material for: VN Thin Films via MOCVD Using a New Vanadium Precursor: Linking Growth Chemistry to Functional Surface Properties
Source: Small Methods. 2025 Dec 18;10(3):e01972. doi: 10.1002/smtd.202501972 (PMC12893275; doi:10.1002/smtd.202501972)
Supplement: Supplementary file 1 — Supporting Information [file SMTD-10-e01972-s001.pdf]

Supporting Information

VN Thin Films via MOCVD Using a New Vanadium Precursor: Linking Growth Chemistry to Functional Surface Properties

*Jean-Pierre Glauber, Julian Lorenz, Ji Liu, Marietta Seifert, Volker Hoffmann, Carlos Abad, Detlef Rogalla, Lars Giebeler, Corinna Harms, Michael Wark, Michael Nolan, Anjana Devi.**

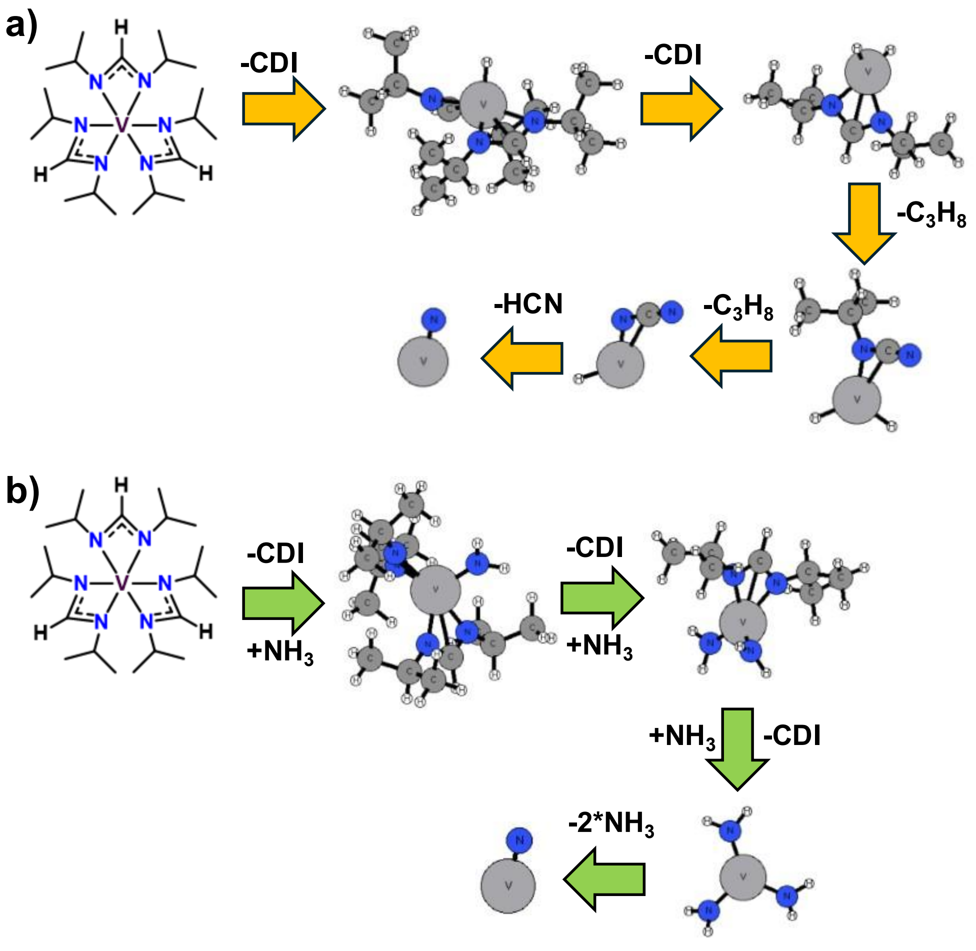

Figure S1. The configurations of key steps along the decomposition pathway in a) absence of NH_3 via loss of CDI-iPr-HCN and b) presence of NH_3. Grey spheres represent carbon, blue spheres represent nitrogen, and white spheres represent hydrogen.

We additionally include the atomic structures of relaxed [V(dpfamd)$_3$] precursor and decomposition products, i.e., the CONTCAR files from VASP calculations.

VN precursor: CONTCAR_VN_precursor

Decomposition with NH_3: CONTCAR_w_R1, CONTCAR_w_R2, CONTCAR_w_R3, CONTCAR_w_R4

Decomposition without NH_3: CONTCAR_R1, CONTCAR_R2, CONTCAR_R3, CONTCAR_R4, CONTCAR_R5

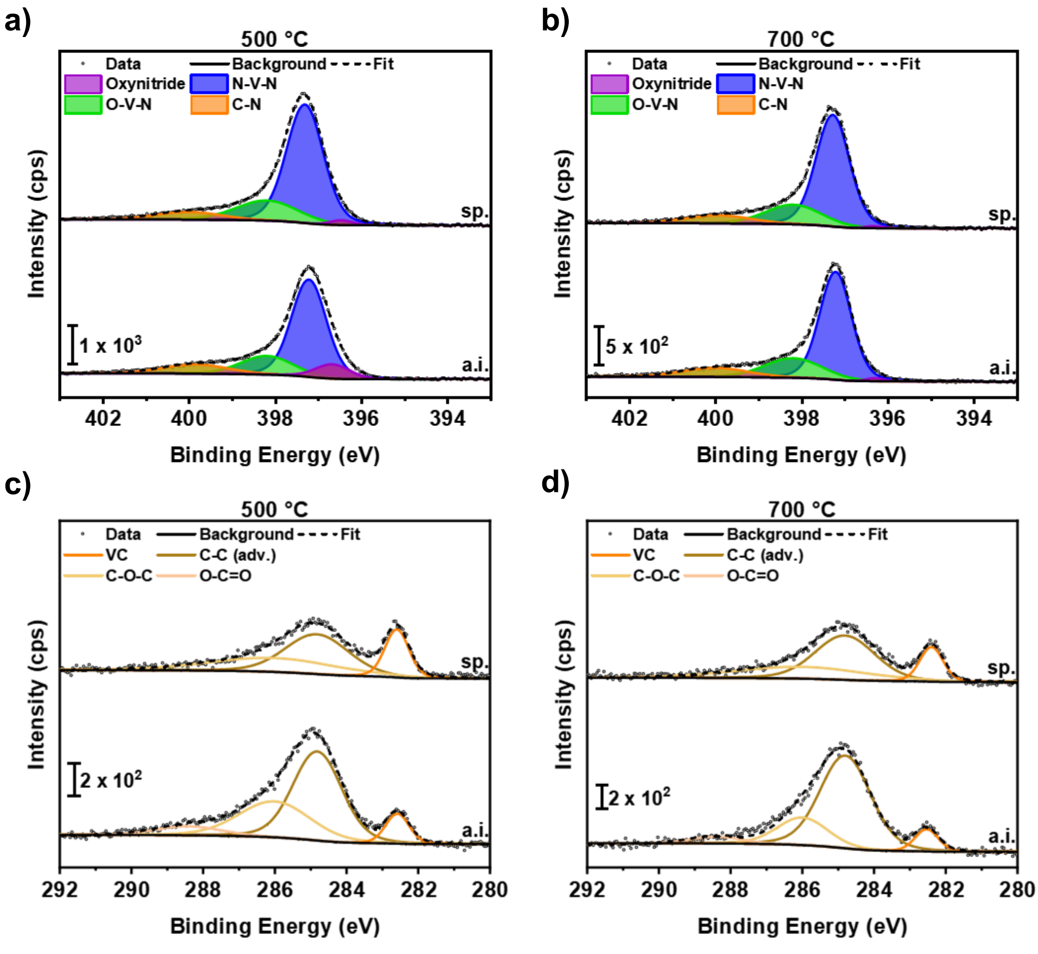

Figure S2. XPS high-resolution core-level spectra of N1s and C1s for VN film on Si grown at (a, c) 500 °C and (b, d) 700 °C. All measurements were conducted on the as-introduced (a.i.) surface and after sputtering (sp.).

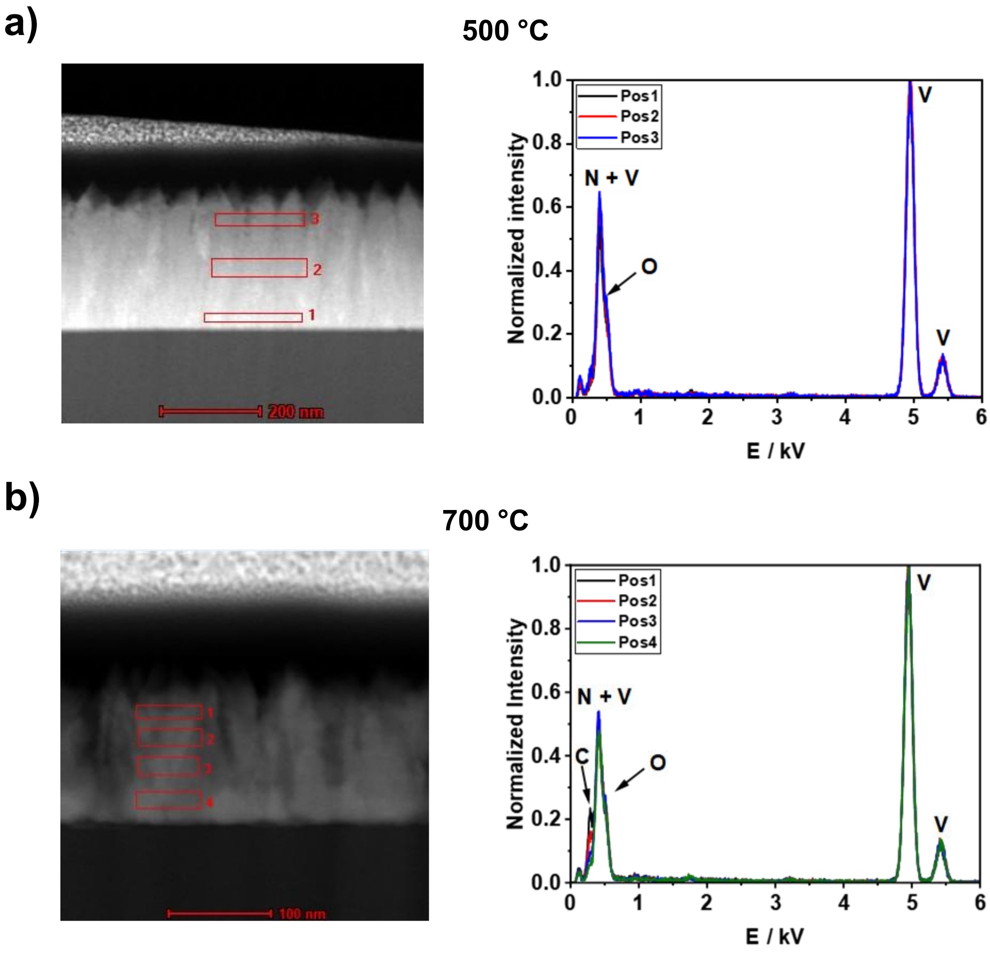

Figure S3. Normalized EDX spectra measured in TEM at different positions of VN grown on Si at a) 500 °C and b) 700 °C.

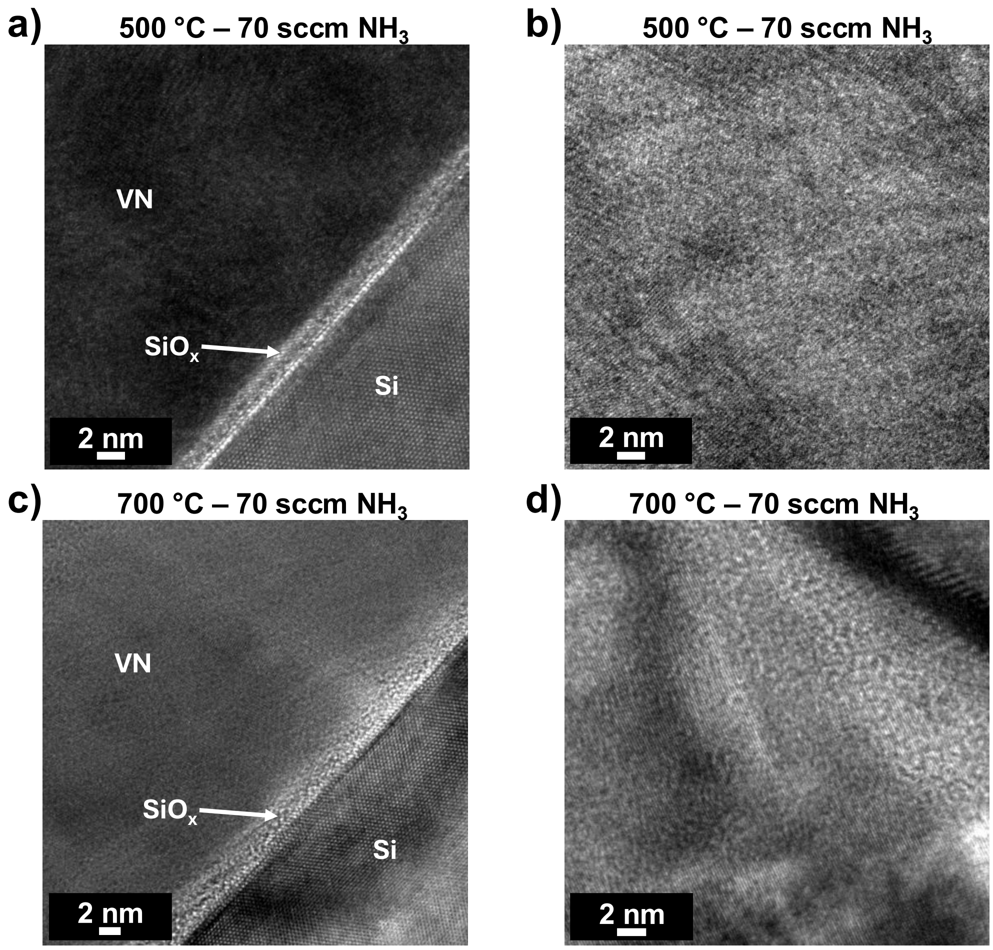

Figure S4. HRTEM images of the interface region between the thin film and substrate, as well as within the VN thin film grown (a, b) at 500 °C and (c, d) at 700 °C, both with an NH_3 flow of 70 sccm.

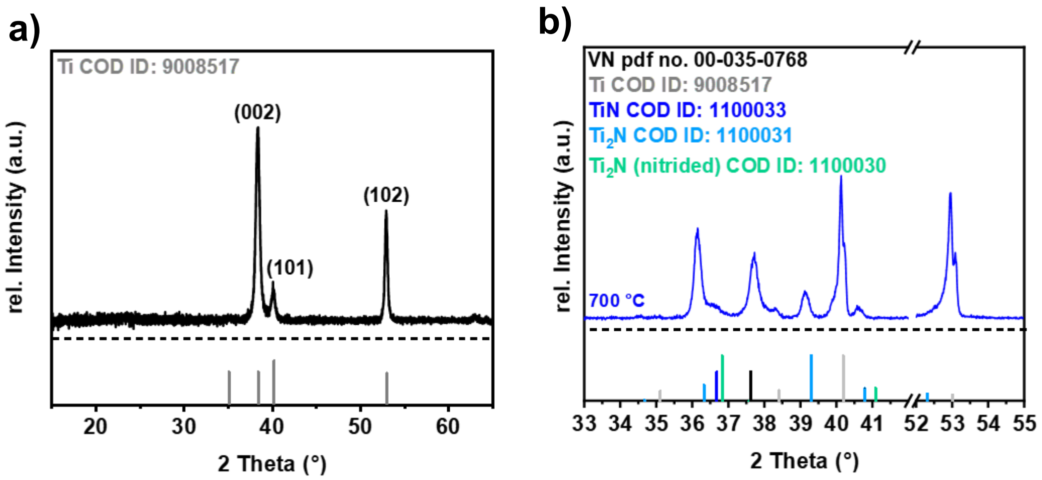

Figure S5. a) XRD pattern of the blank Ti substrate and b) enlarged view of the XRD measurement of the VN thin film grown at 700 °C for 2θ from 33° to 55°. The XRD reference patterns of the different species are shown as follows: cubic VN from the PDF pattern no. 00-035-0768[1] (black), hexagonal Ti COD ID 9008517[2] (grey), cubic TiN COD ID 1100033 (blue),[3] tetragonal Ti_2N COD ID 1100031[4] (cyan), tetragonal Ti_2N (nitrided) COD ID 1100030[5] (green).

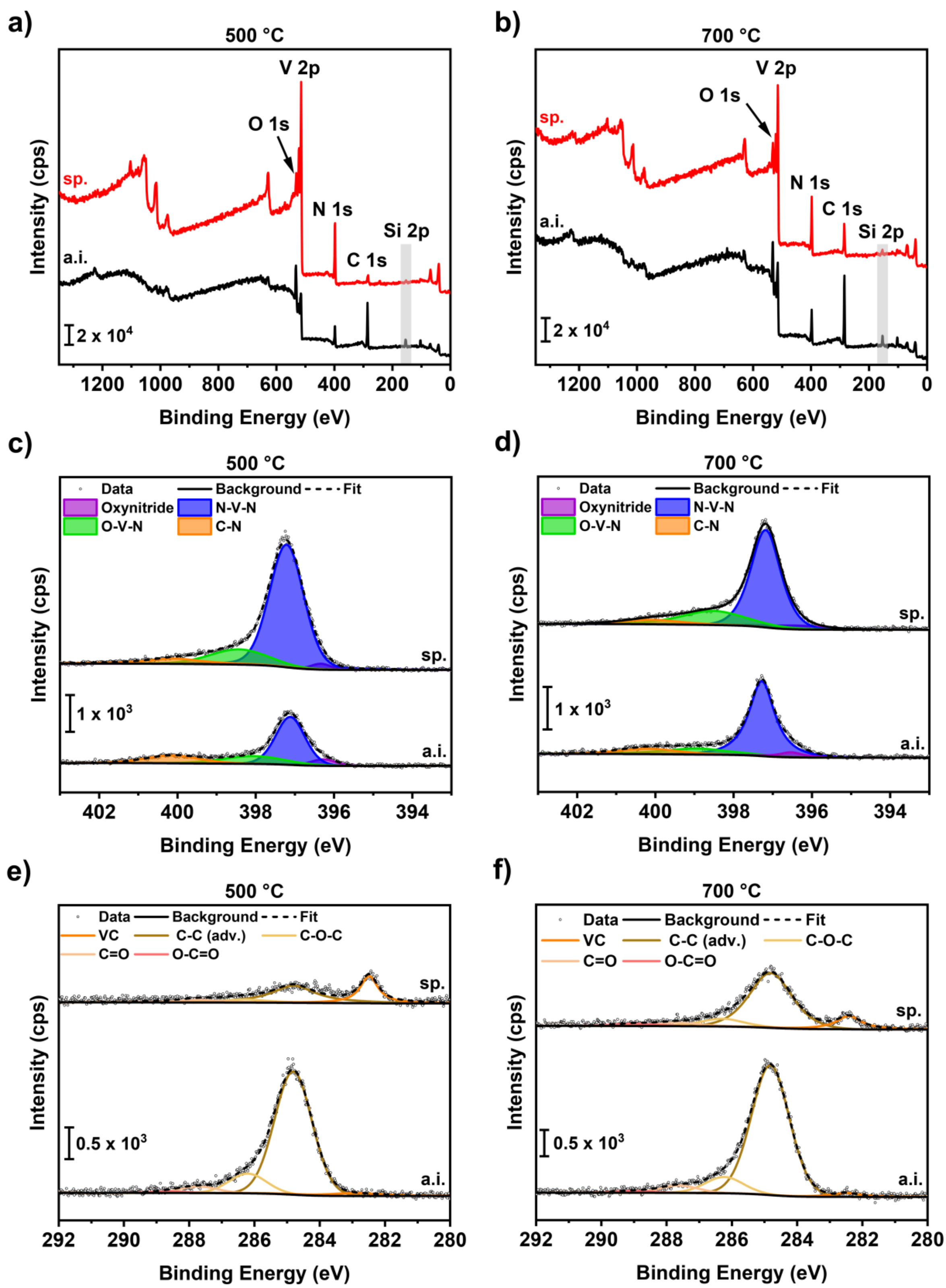

Figure S6. Survey spectra of VN grown at a) 500 °C and b) 700 °C. XPS high-resolution core-level spectra of N 1s and C 1s for VN film on Ti grown at (c, e) 500 °C and at (d, f) 700 °C. All measurements were conducted on the as-introduced (a.i.) surface and after sputtering (sp.).

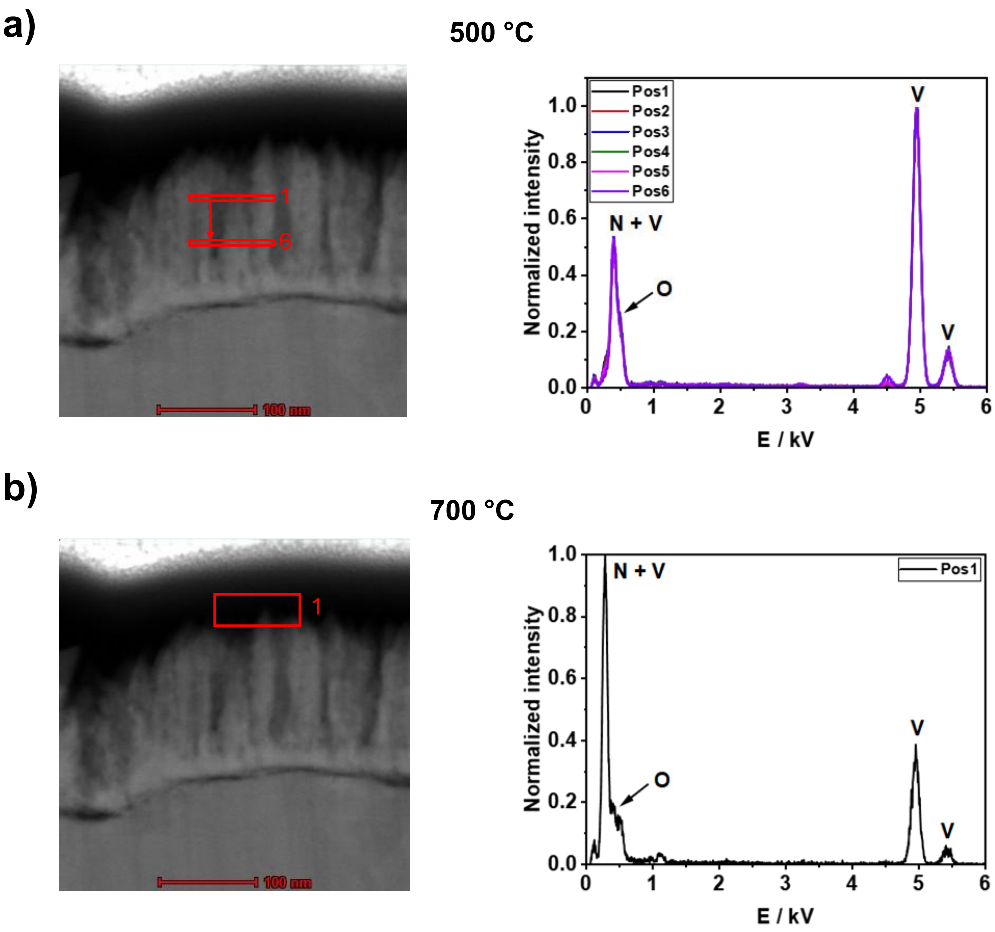

Figure S7. Normalized EDX spectra measured in TEM at a) six different positions in the lower half and b) the surface layer of the VN thin film grown on Ti at 500 °C.

Preliminary eNRR measurements were performed on two selected VN thin film samples deposited on Ti substrates at either 500 °C or 700 °C to demonstrate the capability of the developed thin films for eNRR application. Both samples, VN-500°C and VN-700°C, differ in their crystallinity, whereas the film deposited at 500 °C showed dominant faceting along the (111) plane, while the film at 700 °C showed a polycrystalline structure (Figure 5) instead. The oxygen content is comparable but slightly higher in the VN-500°C (3.9 at%) than in the VN-700°C (1.7 at%) sample. The eNRR activity of specific facets have been theoretically reported with the rock salt (100) facet described as potentially active, while the (111) facet decomposes at eNRR relevant potentials.[6] Also, the incorporation of oxygen in the nitride lattice has been described as beneficial for the eNRR with distinct oxynitride phase as active phase.[7] The developed MOCVD synthesis method here allows the control of both parameters.

The ammonium mass concentration in the electrolyte was negligible small (Figure S8). Ammonium formation after initial CV and activation measurements hints towards contaminants (e.g. nitrates) on the sample surface which were initially reduced to ammonium and excluded from production rates of eNRR turnover experiments. Substantial increase in the ammonium content was observed after d-NRR measurements at -0.2, -0.4 and -0.6 V each for 2 h (equal to 1 h at eNRR potential). The derived production rates, referred to the full 2 h experiments, showed higher ammonium formation in N_2- than Ar-experiments in the case of the VN-700°C sample, with values between 20 and 30 pmol s^{-1} cm^{-2} (Figure S8c). A higher production rate during eNRR compared to background measurements might indicate a possible eNRR activity. However, all measurements have been performed only once, and further experimentation is necessary to prove genuine eNRR activity (see note below). Ammonium formation in Ar-experiments likely originates from protonation of lattice N atoms, which has been described as non-catalytic decomposition in the absence of vacancy replenishment by molecular N_2 for the Mars-van Kreveln mechanism.[8] Thus, further stability evaluation is also necessary which is beyond the scope of this initial study.

Interestingly, the sample VN-500 °C with dominant (111) faceting showed no activity in eNRR experiments. This was expected if following the theoretical predictions by Abghoui et al.[6] where this specific facet was described to decompose under eNRR relevant potentials. Instead, the theoretical active (100) facet, which is present in the VN-700°C sample, showed ammonium formation in our experiments. Thus, our preliminary eNRR work is in alignment with theoretical predictions.

Note that the presented preliminary data cannot prove genuine eNRR activity as repetitions and further experimentation including isotope-labelled $^{15}N_2$ turnover experiments would have been necessary. However, the results provide proof of principle for the studied materials.

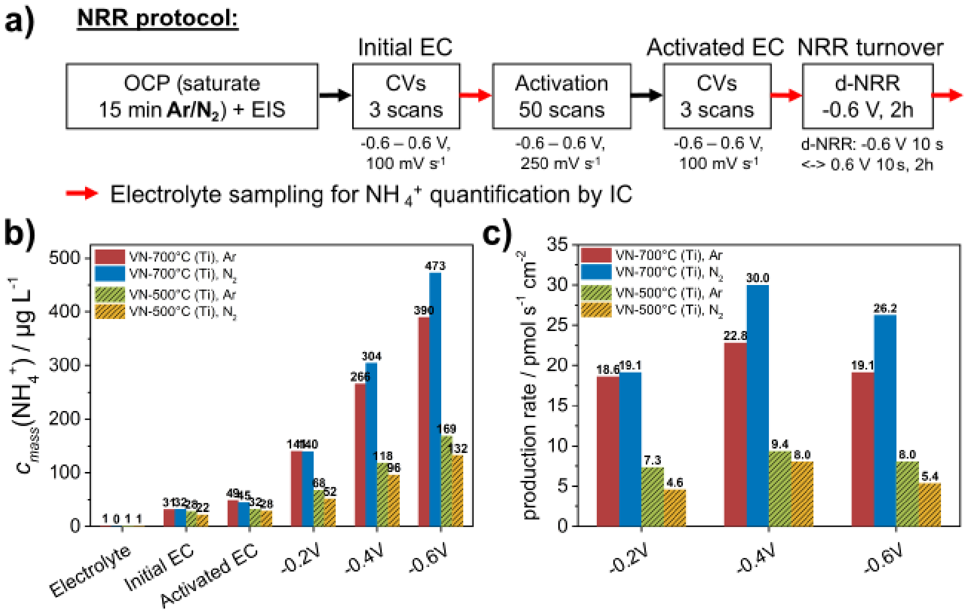

Figure S8. Preliminary eNRR data of developed VN thin films for two selected samples VN-500°C and VN-700°C on Ti substrate a) applied eNRR protocol with highlighting of ion chromatography (IC) sampling b) mass concentration of ammonium (NH_4^+) determined by IC in the freshly prepared electrolyte and after different measurement steps and c) production rates of dynamic eNRR (d-NRR) turnover experiments at varied potentials.

References

1. K. Becker, F. Ebert, *Z. Physik* **1925**, *31*, 268.
2. R. Wyckoff, *Crystal Structure* **1963**, 239.
3. J. R. Ehrlich P, *FIAT Rev. Ger. Sci.* **1948**, 64.
4. B. Holmberg, M. Yhland, R. Dahlbom, J. Sjövall, O. Theander, H. Flood, *Acta Chem. Scand.* **1962**, *16*, 1255.
5. A. N. Christensen, A. Alamo, J. P. Landesman, *Acta Crystallogr., Sect. C: Cryst. Struct. Commun.* **1985**, *41*, 1009.
6. Y. Abghoui, A. L. Garden, J. G. Howalt, T. Vegge, E. Skúlason, *ACS Catal.* **2016**, *6*, 635.
7. X. Yang, J. Nash, J. Anibal, M. Dunwell, S. Kattel, E. Stavitski, K. Attenkofer, J. G. Chen, Y. Yan, B. Xu, *J. Am. Chem. Soc.* **2018**, *140*, 13387.
8. H.-L. Du, T. R. Gengenbach, R. Hodgetts, D. R. MacFarlane, A. N. Simonov, *ACS Sustainable Chem. Eng.* **2019**, *7*, 6839.
